# Transgenerational interactions between pesticide exposure and warming in a vector mosquito

**DOI:** 10.1111/eva.12605

**Published:** 2018-03-05

**Authors:** Tam T. Tran, Lizanne Janssens, Khuong V. Dinh, Robby Stoks

**Affiliations:** ^1^ Evolutionary Stress Ecology and Ecotoxicology University of Leuven Leuven Belgium; ^2^ Institute of Aquaculture Nha Trang University Nha Trang Vietnam; ^3^ National Institute of Aquatic Resources Technical University of Denmark Lyngby Denmark

**Keywords:** carry‐over effects, global warming, multiple stressors, pollution, synergism, transgenerational plasticity, vector control

## Abstract

While transgenerational plasticity may buffer ectotherms to warming and pesticides separately, it remains unknown how combined exposure to warming and pesticides in the parental generation shapes the vulnerability to these stressors in the offspring. We studied the transgenerational effects of single and combined exposure to warming (4°C increase) and the pesticide chlorpyrifos on life‐history traits of the vector mosquito *Culex pipiens*. Parental exposure to a single stressor, either warming or the pesticide, had negative effects on the offspring: parental exposure to both warming and the pesticide resulted in an overall lower offspring survival, and a delayed offspring metamorphosis. Parental exposure to a single stressor did, however, not alter the vulnerability of the offspring to the same stressor in terms of survival. Parental pesticide exposure resulted in larger offspring when the offspring experienced the same stressor as the parents. Within both the parental and offspring generations, warming made the pesticide more toxic in terms of survival. Yet, this synergism disappeared in the offspring of parents exposed to both stressors simultaneously because in this condition, the pesticide was already more lethal at the lower temperature. Our results indicate that transgenerational effects will not increase the ability of this vector species to deal with pesticides in a warming world. Bifactorial transgenerational experiments are crucial to understand the combined impact of warming and pesticides across generations, hence to assess the efficacy of vector control in a warming world.

## 1 INTRODUCTION

A crucial factor that will determine the outcome of pest control programs in a warming world is whether vector species will change their tolerance to pesticides and to warming across generations. While it is well documented that this can happen through rapid evolution (e.g., Hemingway, Field, & Vontas, 2002; Koella, Saddler, & Karacs, 2012; Liu, 2015), this is much less studied for the potentially more rapid nongenetic changes in tolerance due to transgenerational, plastic effects (Hariprasad & Shetty, 2017; Prud'homme, Chaumot, Cassar, David, & Reynaud, 2017). Both mechanisms are linked as adaptive phenotypic plasticity may buy additional time for adaptation to occur and may provide a mechanism for adaptation to occur rapidly (Ghalambor, McKay, Carroll, & Reznick, 2007; Stoks, Govaert, Pauwels, Jansen, & De Meester, 2016).

Transgenerational effects occur through nongenetic parental effects, whereby environmental conditions experienced by the parental generation influence the phenotype of subsequent generations. Both maternal (e.g., Shama, Strobel, Mark, & Wegner, 2014; Storm & Lima, 2010) and paternal effects (e.g., Bonduriansky & Day, 2009; Bonduriansky & Head, 2007) have been described. Mechanisms of nongenetic inheritance that alter the phenotypes of offspring include maternal and paternal provisioning (Curley, Mashoodh, & Champagne, 2011) such as the transfer of nutrients from mother to offspring, epigenetic changes (Bonduriansky, Crean, & Day, 2012; Ho & Burggren, 2010; Munday, 2014) and gamete plasticity (Jensen, Allen, & Marshall, 2014).

Transgenerational effects have been described both in response to warming (e.g., Munday, 2014; Salinas & Munch, 2012; Shama et al., 2014) and to pesticide exposure (e.g., Brausch & Salice, 2011; Kim, Yu, Jeong, & Kim, 2014) and raise the concern whether we can reliably predict the biological impact of these stressors based on single‐generation experiments (Kim et al., 2014; Yu, Zhang, & Yin, 2016). Indeed, transgenerational effects can make offspring both more (e.g., Pölkki, Kangassalo, & Rantala, 2012; Schultz et al., 2016) or less (e.g., Brausch & Salice, 2011; Kim et al., 2014; Reátegui‐Zirena et al., 2017) vulnerable to stressors compared to the parental generation. The emerging view based on recent empirical studies on warming is that transgenerational plasticity may buffer the negative effects of warming on ectotherms (Munday, 2014; Shama et al., 2014), yet this may be biased because of methodological weaknesses in the design of the studies (Kielland, Bech, & Einum, 2017).

An important phenomenon when assessing the impact of pesticides in a warming world is that many pesticides become more toxic under warming (Holmstrup et al., 2010; Liess, Foit, Knillmann, Schäfer, & Liess, 2016; Noyes & Lema, 2015; Noyes et al., 2009), making pest control potentially more efficient. Yet, no studies have explored how combined exposure to warming and pesticides in the parental generation shapes the vulnerability to these stressors in the offspring. Such studies are much needed to address key questions relevant for pest control such as whether the typical synergistic effects between both stressors bridge generations and if so whether the synergism is modulated when offspring are exposed to the same stressor combination. More generally, transgenerational experiments typically considered one stressor and none manipulated two stressors in a full factorial way in both the parental and the offspring generations limiting our insight in how effects of stressor interactions change across generations.

We investigated the transgenerational effects of single and combined warming and pesticide exposure on a vector mosquito where both the parental and the offspring generations were exposed to both stressors in a full factorial design. We addressed the following three questions about transgenerational effects: Does parental exposure to warming and/or to the pesticide affect (Q1) the condition of the offspring (irrespective of stressors experienced in the offspring generation), (Q2) the ability of the offspring to deal with the same stressor as their parents, and (Q3) the expected synergism between warming and pesticide exposure in the offspring.

As model species, we studied the vector mosquito *Culex pipiens* biotype *molestus* (Forskål, 1775) (hereafter called *Culex pipiens*). Species of the *Culex* complex are vectors of several viruses and pathogens such as West Nile and St. Louis encephalitis viruses, avian malaria and filarial worms (Farajollahi, Fonseca, Kramer, & Kilpatrick, 2011). Studying the combined effect of warming and pesticide exposure is especially relevant in *Culex pipiens*, the primary vector for West Nile Virus (WNV), a pathogen of global concern. This is because the invasion and transmission of WNV is expected to increase with increasing temperature (Kilpatrick, Meola, Moudy, & Kramer, 2008; Paz, 2015), making it crucial to investigate the efficacy of the pesticide‐based control of its primary vector under warming. *Culex pipiens* is the most common vector mosquito species in urban areas in Europe and the USA (Fonseca et al., 2004; Paz, 2015), therefore being the target of many vector control campaigns (Kilpatrick, 2011). As pesticide, we chose the organophosphate insecticide chlorpyrifos (CPF), one of the most frequently used pesticides worldwide in pest control programs including mosquito larvae control (Eaton et al., 2008). Notably, previous studies on other aquatic insects showed the toxicity of chlorpyrifos to be magnified under warming (e.g., Lydy, Belden, & Ternes, 1999; Van Dinh, Janssens, Debecker, & Stoks, 2014).

## 2 MATERIALS AND METHODS

A laboratory culture of *C. pipiens* was started from a stock culture at the Helmholtz Centre for Environmental Research—UFZ, Germany. This stock culture was previously initiated from field collected egg rafts (see Tran, Janssens, Dinh, Op de Beeck, & Stoks, 2016; Appendix S1). The mosquito culture was housed in a climate‐controlled room at 20°C with a photoperiod of 14:10‐hr light:dark and a humidity of 70 ± 10%. The culture was acclimated in the laboratory for >10 generations before starting the experiment. The *C. pipiens* biotype *molestus* can lay a single batch of eggs without a blood meal (Fonseca et al., 2004). We therefore did not provide the adults with a blood meal to ensure that all the egg rafts used in both generations were the first clutches of each female thereby controlling for potential inter‐raft changes in egg quality.

### 2.1 Experimental design

**Figure 1:**
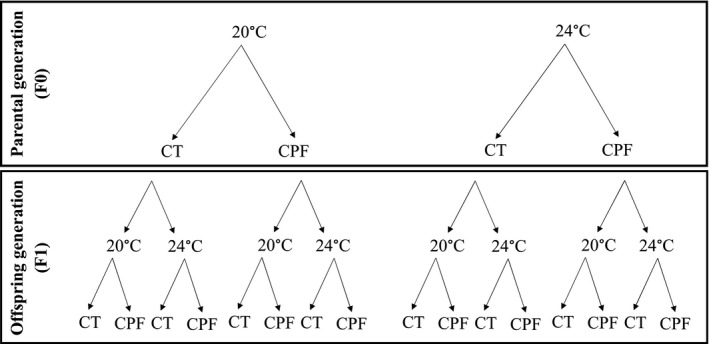
Bifactorial crossed design for testing transgenerational effects of warming and pesticide exposure on mosquitoes with four treatment combinations in the parental generation (F0) and sixteen treatment combinations in the offspring generation (F1). CT = solvent control, CPF = 0.23 μg/L chlorpyrifos

To investigate the transgenerational effects of warming and pesticide exposure, we carried out a full factorial experiment for two generations. In the first, parental generation (F0) larvae were exposed to one of the four treatment combinations (2 temperatures × 2 pesticide treatments). In the second, offspring generation (F1) larvae produced by each treatment combination in the first generation were randomly allocated to each of the four temperature‐by‐pesticide treatment combinations as in the first generation. This resulted in 16 treatment combinations in the second generation (Figure 1). In each generation, mosquitoes were continuously exposed to the temperature treatment from the egg stage until the adult stage, while the pesticide exposure occurred during 5 days in the final larval stage (L4). Based on the guidelines by WHOPES (2005), we exposed larvae in the L4 stage in groups of 30. The exposure time was set at 5 days as at day 6, the first larvae pupated in a pilot experiment. The two rearing temperatures chosen, 20 and 24°C, represent the current mean summer water temperature of ponds where the mosquito culture originates (see Tran et al., 2016; Appendix S1), and the expected mean temperature by 2100 under the 4°C warming scenario RCP 8.5 (IPCC 2013), respectively.

To select the chlorpyrifos concentration for the exposure experiment, we first ran a range finding experiment where we tested following range of concentrations: 0 (solvent control), 0.05, 0.1, 0.15, 0.18, 0.20, 0.23, 0.25, 0.30 and 0.50 μg/L. The stock solution of chlorpyrifos (CPF) was prepared in absolute ethanol at a concentration of 500 μg/ml and was stored at 4°C in the dark to avoid degradation. All concentrations, including the solvent control, contained a similar amount of ethanol (0.46 μl/L). We exposed the larvae for 5 days, with a pulse at day 1 and after 48 hr at day 3. We applied the second pulse to minimize differences in CPF concentrations between both temperatures. A nominal concentration of 0.23 μg/L CPF was chosen because it induced low (9.4% at 20°C) mortality during the exposure period, which gave the opportunity to see delayed effects in the second generation and to identify synergistic interactions with temperature. The measured concentration in the experimental vials (based on a pooled sample) was 0.27 μg/L (quantified using LC‐MS/MS). The recommended application dose of CPF for control of mosquito larvae in open water bodies is 1.1–2.5 mg/m (WHO 2017). If we assume the average depth of the treated water bodies is ca. 0.5–1 m (matching typical shallow ponds and lakes inhabited by mosquito larvae), the recommended application dose will result into a pulse concentration in the water bodies of 1.1–5 μg/L. In natural water, there is an initial rapid decline of CPF, but the remaining CPF fraction stays long in the water (after 10 days still 3%) (Mazanti et al., 2003). Hence, applying one single pulse of the recommended dose is expected to result after 5 days in exposure concentrations similar to the one we here applied.

### 2.2 Experimental procedure

A detailed scheme of the experimental procedure is shown in Figure S1 (Appendix S1). At the start of the experiment, 108 egg rafts were individually incubated in 200‐ml glass vials filled with 125 ml of dechlorinated tap water in one of two climate‐controlled rooms at 20 or 24°C. During the larval stage, mosquitoes were fed with a mixture of Supradyn^®^ vitamins (3%), Olvarit^®^ 7 cereal flakes (46%) and wheat germs (51%) (0.313 mg per larva, Tran et al., 2016).

Three days after hatching (when most larvae at both temperatures were in their second instar), mosquito larvae were placed in the same type of vials in groups of 40. Each initial vial contained larvae that hatched from a single, unique egg raft. We started with 108 initial vials in the parental (F0) generation, and with 432 initial vials in the offspring (F1) generation (Figure S1). When larvae entered the pesticide exposure period, they were transferred to the same type of vials filled with 125 ml of pesticide or ethanol solvent medium. Mortality prior to exposure was minor (ca. 3%). In each vial, we placed a set of larvae that had moulted to the L4 stage within the last 24 hr. To obtain enough synchronized larvae to start the pesticide exposure, we pooled larvae from three initial vials of the same temperature treatment and redistributed them to install two exposure vials of 30 larvae (one control exposure vial and one exposure vial with CPF). This resulted in 18 exposure vials per treatment combination (total of 72 vials in F0, 288 vials in F1). The groups of three initial vials of the same temperature treatment that were pooled are referred to as subsets; two subsets of six vials of the same temperature are named a set (see Figure S1, Appendix S1). From each set, we made two control exposure vials and two CPF exposure vials.

After a 5‐day pesticide exposure period (with refreshment of the medium on day 3), all larvae were transferred to vials with clean water until pupation. Pupae were daily collected and transferred to 30‐ml plastic cups filled with 10 ml of clean water. Each cup was placed in a small insectary (8 × 10 × 15 cm^3^) to house the mosquitoes after metamorphosis. To obtain enough eggs, pupae arising from two exposure vials of the no‐pesticide control treatment of the same set were housed in the same no‐pesticide control insectary for oviposition. The same was carried out for the pupae of the pesticide treatment. This resulted in nine replicate insectaries per treatment combination, with in total 36 insectaries in F0, and 144 insectaries in F1 (see Figure 1, Appendix S1). Each insectary was provided with a paper filter soaked in a 6% glucose solution, which was replaced every other day, for feeding and a small plastic cup filled with water for oviposition.

To start the F1 generation, we daily checked for new egg rafts and immediately divided them equally amongst the two temperature treatments. In total, we used 432 egg rafts to start the second generation. Each egg raft was hatched individually in a separate vial. After hatching, the larvae of the F1 generation underwent the same experimental procedure as described above. From each insectary, 12 initial vials were started, a set of six vials at 20°C and a set of six vials at 24°C (see Figure S1 in Appendix S1).

### 2.3 Response variables

In both generations, we scored survival and development time to metamorphosis and size of the adults at metamorphosis. We scored survival in each vial from the start of the pesticide exposure period until metamorphosis into the adult stage. In addition, we quantified survival across the 5‐day larval exposure period; this response variable is reported in the supplementary material in Appendix S2. The development time was calculated for each surviving larva as the number of days from the start of the L4 stage until adult metamorphosis. Given the large numbers of mosquitoes emerging synchronously, we did not identify the sex of the animals at this moment. At the end of the experiment, we calculated the sex ratio based on the total number of males and females that emerged per insectary. To estimate size at metamorphosis, we measured the wing length of the adults (Huestis et al., 2011). We daily collected dead adults from each insectary and stored these in Eppendorf tubes. At the end of the experiment, wings of five males and five females per insectary were photographed using a microscope (SZX 16, Olympus, Japan) connected with a digital camera (Basler AG, Ahrensburg, Hamburg, Germany) and controlled by the program Streampix v.3.55.0 (NorPix Inc., Montreal, QC, Canada). Wing length was measured as the distance between the alular notch and the intersection of the radius 3 vein and the outer margin based on the protocol of Huestis et al. (2011) using the computer program ImagePro Plus v.5.0.0.39 (Media Cybernetics, Bethesda, MD, USA). As the results on sex differences are not the focus of our study, we present them in Appendix S3.

### 2.4 Statistical analyses

All statistical analyses were performed in R v3.4.0 (R Development Core Team, 2017) with the packages lme4 (v1.1‐14) (Bates, Maechler, Bolker, & Walker, 2015), car (v2.1‐5) (Fox & Weisberg, 2011), effects (v2.27‐2) (Fox, 2003) and lsmeans (v2.26‐3) (Lenth, 2016). We tested for effects of stressors (temperature and pesticide) in the parental (F0) and/or in the offspring (F1) generations on the response variables (survival, sex ratio, development time and size at metamorphosis) using separate linear mixed models. We did not simplify the models; instead, we kept and report the full models. The significance of the explanatory variables was determined using Wald chi‐square tests.

Survival of each adult was scored as 1 (alive) and 0 (dead). Sex of each adult was scored as 1 (male) and 0 (female). When analysing the effects on survival (to metamorphosis) and sex ratio in both generations, we used generalized linear mixed models with a binomial error structure and the logit link function. To take into account groups of larvae were from the same exposure vial and the experimental procedure (pooling and remixing of larvae), we added the appropriate random factors to the models. In the parental generation, insectary‐F0 (the insectary of adults in the F0 generation) nested in the set‐F0 and set‐F0 were added to the model as random factors. In the offspring generation, insectary‐F1 nested in set‐F1, set‐F1 nested in insectary‐F0 and insectary‐F0 were included in the model as random factors (see Appendix S1).

For analysing the effects on development time and size at metamorphosis, linear mixed models were used. For both the development time and size, we added as random factors insectary‐F0 nested in set‐F0 and set‐F0 when analysing the parental generation, while we added insectary‐F1 nested in set‐F1, set‐F1 nested in insectary‐F0 and insectary‐F0 when analysing the offspring generation.

## 3 RESULTS

### 3.1 Within‐generation effects of temperature and pesticide exposure in the parental (F0) generation

**Figure 2:**
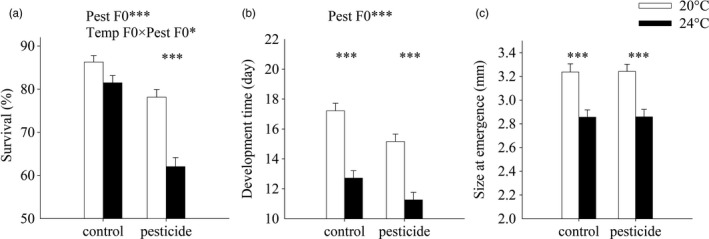
Survival to metamorphosis (a), development time (b) and size at emergence (c) of *C. pipiens* mosquitoes in the parental generation as a function of temperature and pesticide treatments. These response variables are based on nine replicated insectaries per original treatment combination. Given are LS‐means with 1 SE. The asterisks indicate significant effects of warming for a given pesticide treatment (*p <* *.05, **p <* *.01, ***p <* *.001)

In the parental generation, survival to metamorphosis was ca. 86% at 20°C in the solvent control. Survival was negatively affected by warming and especially by CPF exposure (Table 1, Figure 2A). Moreover, the effect of CPF was stronger under warming: while the pesticide reduced survival ca. 8% at 20°C, it reduced survival ca. 20% at 24°C (Temp F0 × Pesticide F0 interaction, Table 1, Figure 2A). The sex ratio was not affected by warming or CPF exposure or the interaction between the two stressors (Table S1, Figure S5).

**Table 1:** Effects of temperature and pesticide exposure on survival to metamorphosis, development time and size at emergence of *Culex pipiens* mosquitoes in the parental (F0) generation

Effect	Survival			Development time			Size at emergence		
	*df*	Wald χ^2^	*p*	*df*	Wald χ^2^	*p*	*df*	Wald χ^2^	*p*
Temperature F0	1	27.95	**<.001**	1	40.52	**<.001**	1	113.69	**<.001**
Pesticide F0	1	51.93	**<.001**	1	35.42	**<.001**	1	0.03	.855
Sex							1	1,718.83	**<.001**
Temperature F0 × Pesticide F0	1	3.87	**.049**	1	1.10	.295	1	0.01	.932

Significant *p* values (*p *<* *.05) are indicated in bold.

Mosquito larvae emerged ca. 26% (ca. 4.5 days) earlier at 24°C and ca. 12% (ca. 2 days) earlier when exposed to CPF (Table 1, Figure 2B). There was no interaction between the temperature and the pesticide treatment (Table 1). Animals reared at 24°C emerged at a smaller size (Figure 2C), while the size was not affected by the pesticide treatment or the interaction between the two stressors (Table 1).

### 3.2 Within‐ and transgenerational effects of temperature and pesticide exposure in the offspring (F1) generation

**Figure 3:**
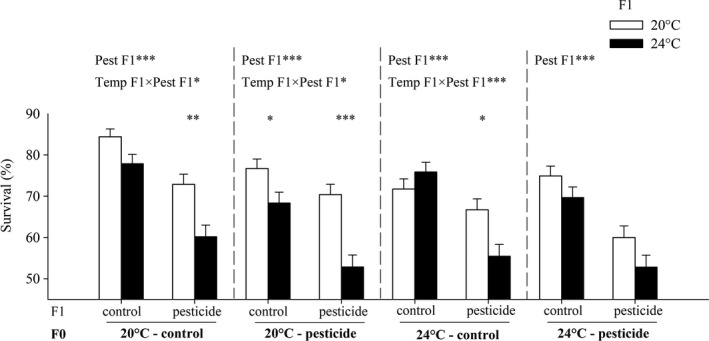
Survival to metamorphosis of *C. pipiens* mosquitoes in the offspring generation as a function of temperature and pesticide treatments in the parental (F0) and offspring (F1) generations. Survival is based on nine replicated insectaries per original treatment combination. Given are LS‐means with 1 SE. The asterisks indicate significant effects of warming for a given pesticide treatment (*p <* *.05, **p <* *.01, ***p <* *.001)

Overall, parental exposure to both warming and CPF reduced survival in the offspring, irrespective of the treatment experienced by the offspring (main effects Temp F0 and Pest F0, Table 2, Figure 3). Also in the offspring generation, both warming and especially exposure to CPF reduced survival (main effects of Temperature F1 and Pesticide F1, Table 2, Figure 3). As in the first generation, the negative effect of CPF was overall stronger under warming (Temp F1 × Pest F1, Table 2). Yet, this was not the case when the parents had been exposed to CPF at 24°C: these offspring already showed a strong CPF‐induced reduction in survival at 20°C (ca. 16%) and no further reduction at 24°C (Temp F0 × Pest F0 × Temp F1 × Pest F1, Table 2, Figure 3). For the other three combinations of parental temperature and parental pesticide treatment, the average survival reduction caused by the pesticide was ca. 7% at 20°C and ca. 18% at 24°C (Figure 3). No significant effects of the stressors or their interactions on the sex ratio were detected (Table S2, Figure S6).

**Table 2:** Effects of temperature and pesticide exposure during the parental (F0) and offspring (F1) generations on survival to metamorphosis, development time and size at emergence of *Culex pipiens* mosquitoes in the offspring (F1) generation

Effect	Survival			Development time			Size at emergence		
	*df*	Wald χ^2^	*p*	*df*	Wald χ^2^	*p*	*df*	Wald χ^2^	*p*
Temperature F0 (Temp F0)	1	10.93	**<.001**	1	10.37	**.001**	1	2.40	.122
Pesticide F0 (Pest F0)	1	9.73	**.002**	1	2.23	.135	1	0.27	.603
Temperature F1 (Temp F1)	1	37.91	**<.001**	1	550.49	**<.001**	1	752.62	**<.001**
Pesticide F1 (Pest F1)	1	107.34	**<.001**	1	50.24	**<.001**	1	18.32	**<.001**
Sex							1	11,091.99	**<.001**
Temp F0 × Pest F0	1	1.65	.199	1	0.09	.763	1	2.61	.106
Temp F0 × Temp F1	1	7.64	**.006**	1	4.20	**.040**	1	2.70	.100
Pest F0 × Temp F1	1	0.73	.393	1	9.23	**.002**	1	0.06	.814
Temp F0 × Pest F1	1	0.18	.673	1	1.90	.168	1	2.30	.129
Pest F0 × Pest F1	1	0.55	.457	1	1.42	.233	1	6.34	**.012**
Temp F1 × Pest F1	1	6.63	**.010**	1	0.46	.498	1	1.53	.215
Temp F0 × Pest F0 × Temp F1	1	0.03	.870	1	0.11	.743	1	0.14	.705
Temp F0 × Pest F0 × Pest F1	1	2.77	.096	1	3.54	.060	1	2.16	.142
Temp F0 × Temp F1 × Pest F1	1	0.13	.717	1	0.04	.845	1	0.06	.814
Pest F0 × Temp F1 × Pest F1	1	1.14	.286	1	0.09	.761	1	0.05	.823
Temp F0 × Pest F0 × Temp F1 × Pest F1	1	3.99	**.046**	1	0.08	.771	1	0.21	.644

Significant *p* values (*p *<* *.05) are indicated in bold.

**Figure 4:**
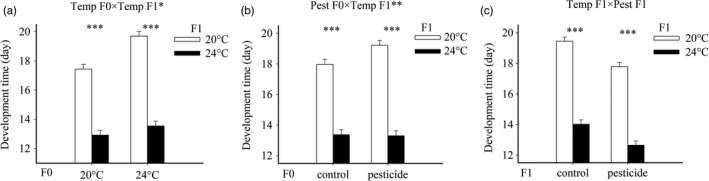
Development time of *C. pipiens* mosquitoes in the offspring generation as a function of parental rearing temperature and offspring rearing temperature (a), parental pesticide exposure and offspring rearing temperature (b), and offspring rearing temperature and offspring pesticide exposure (c). Development times are based on nine replicated insectaries per original treatment combination. The asterisks indicate significant effects of warming for a given temperature/pesticide treatment (*p <* *.05, **p <* *.01, ***p <* *.001)

As in the parental generation, development time was reduced by ca. 28% (ca. 5 days) under warming and by ca. 9% (ca. 1.5 days) under CPF exposure (main effects Temperature F1 and Pesticide F1, Table 2, Figure 4C and Figure S4). Metamorphosis was also delayed in response to parental warming (ca. 10%) and parental pesticide exposure (ca. 7%), but only in offspring that were reared at 20°C (Temp F0 × Temp F1 and Pest F0 × Temp F1, Table 2). In the offspring reared at 24°C, these percentages were 5% and −0.6%, respectively, and nonsignificant.

**Figure 5:**
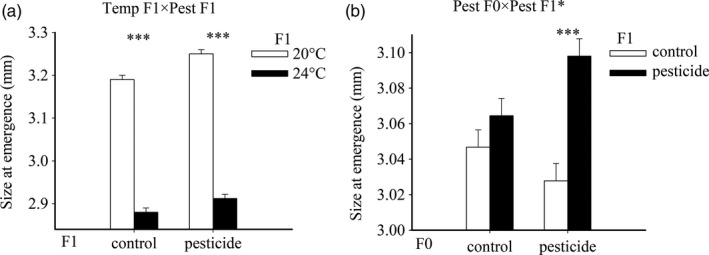
Offspring size at emergence of *C. pipiens* mosquitoes as a function of offspring rearing temperature and offspring pesticide exposure (a), and parental pesticide exposure and offspring pesticide exposure (b). Body sizes are based on nine replicated insectaries per original treatment combination. Given are LS‐means with 1 SE. The asterisks indicate significant differences between the treatment levels associated with the coupled bars (*p <* *.05, **p <* *.01, ***p <* *.001)

Also in the offspring generation, mosquitoes reared at 24°C emerged at a smaller size (Table 2, Figure 5A). To a lesser extent, size was also influenced by the stressors experienced by the parents. Parents exposed to CPF had ca. 3% larger offspring when offspring were exposed to CPF compared to only 0.3% when parents had been exposed to the solvent control (Pest F0 × Pest F1, Table 2, Figure 5B).

## 4 DISCUSSION

The within‐generation effects of warming and pesticide exposure were similar for both generations. Consistent with our expectations that both warming and pesticide exposure are stressful and interact synergistically, they reduced survival and the lethal effect of chlorpyrifos was stronger under warming. Transgenerational effects of parental rearing temperature and pesticide exposure were common and moderately interacted with the effects of these stressors in the offspring generation. In general, we detected in the offspring costs rather than beneficial effects in response to exposure to a stressor in the parental generation. Notably, joint exposure of the parents to both warming and the pesticide made the offspring more vulnerable to the pesticide, resulting in the loss of the synergism between warming and pesticide exposure.

### 4.1 Within‐generation effects of temperature and pesticide exposure

In both generations, both warming and chlorpyrifos exposure reduced survival and did so in a synergistic way. A higher mortality under the here applied mild warming has been observed in the study species (Ciota, Matacchiero, Kilpatrick, & Kramer, 2014; Tran et al., 2016). It confirms the previously reported pattern of local thermal adaptation given that 20°C corresponds to the mean summer temperature of the here used mosquito source population (Tran et al., 2016). The increased toxicity of chlorpyrifos under warming is expected (Noyes et al., 2009) due to the higher uptake and the accelerated biotransformation of this pesticide to more toxic o‐analog metabolites at higher temperature (Buchwalter, Jenkins, & Curtis, 2003; Lydy et al., 1999), combined with a reduced condition of the mosquitoes under warming. The higher uptake of chlorpyrifos may have been further increased because of the smaller size under warming (Buchwalter, Jenkins, & Curtis, 2002; Rubach et al., 2012). Another explanation could be a lower allocation of resources to detoxification in larvae reared at 24°C who may have invested more resources in the accelerated development to escape the stressful environment. Note that we kept the pesticide concentrations constant at both temperatures while in nature chlorpyrifos may degrade faster at higher temperatures thereby buffering its higher toxicity under warming (Op de Beeck, Verheyen, Olsen, & Stoks, 2017). Future studies would therefore benefit from also including a treatment, where the pesticide is allowed to degrade in a temperature‐dependent way.

The effects of warming and pesticide exposure carried over to the adult stage, with larvae reared at the high temperature metamorphosing earlier and at a smaller size, and pesticide‐exposed animals metamorphosing earlier. Accelerated development resulting in a smaller size is a well‐known response to warming (temperature‐size rule, Atkinson, 1994) and has been reported in *Culex* mosquitoes, including the study species (Ciota et al., 2014). In contrast to warming, which strongly accelerated the development with ca. 26% (ca. 4.5 days), pesticide exposure only resulted in a ca. 12% accelerated development (ca. 2 days). This together with the low exposure concentration, and the short exposure duration (5 days) used in this experiment may explain the lack of a pesticide effect on adult size. An accelerated development induced by pollutant exposure has also been documented in other mosquitoes (e.g., Prud'homme et al., 2017).

### 4.2 Transgenerational effects of temperature and pesticide exposure

#### 4.2.1 (Q1) Does parental exposure to warming and/or to a pesticide affect the overall condition of the offspring (irrespective of stressors in the offspring generation)?

Parental exposure to a single stressor, being warming or the pesticide, had negative effects in the offspring irrespective of the stressors experienced by the offspring (main effects of parental warming and parental pesticide exposure in Table 2). Parental exposure either to warming or to the pesticide resulted in a lower offspring survival and delayed offspring metamorphosis (the later effect only observed when offspring were reared at the nonstressful 20°C). This suggests that stressed parents produced lower quality offspring, potentially caused by alteration in egg yolk content (Corrales, Thornton, White, & Willett, 2014; Hahn, Schenk, & Schulz, 2002) and DNA damage in the eggs (Guillaume, Monro, & Marshall, 2016). This transgenerational cost of parental warming contrasts with the studies reporting adaptive transgenerational effects of warming (e.g., reviewed in Donelson, Salinas, Munday, & Shama, 2018). Yet, our results are consistent with other findings (both on vertebrates and invertebrates) that demonstrated negative effect of a higher paternal rearing temperature on offspring survival (e.g., Guillaume et al., 2016; Shama & Wegner, 2014), and development (Ferrer, Dorn, & Mazzi, 2013; Walsh, Whittington, & Funkhouser, 2014). These differences may be partially associated with the magnitude of the warming applied with a smaller gradual increase being more likely to result in adaptive transgenerational effects (Donelson, Wong, Booth, & Munday, 2016). Similarly, sublethal transgenerational costs of exposure to pesticides or other toxicants have been documented in both invertebrates (e.g., Kimberly & Salice, 2015; Schultz et al., 2016; Yu & Liao, 2016) and vertebrates (e.g., Bhandari, vom Saal, & Tillitt, 2015). The underlying mechanisms are unknown, but epigenetic processes have been suggested to play a role (Schultz et al., 2016).

#### 4.2.2 (Q2) Does parental exposure to warming and/or to a pesticide affect the ability of the offspring to deal with the same stressor?

Parental exposure to a single stressor, being warming or the pesticide, did not change the vulnerability of the offspring when the offspring were exposed to the same stressor in terms of survival and development time, but did so for size (absence/presence of interactions between parental exposure and offspring exposure to the same stressor in Table 2). For warming, this contrasts with recent studies which showed that parental exposure to warming reduced the negative effects of warming in the offspring (Chakravarti et al., 2016; Donelson et al., 2016; Salinas & Munch, 2012; Shama et al., 2014). Yet, also the opposite pattern has been observed with maternal exposure to warming resulting in a lower offspring survival under warming (Guillaume et al., 2016; Shama & Wegner, 2014). Again, these differences may be due to the magnitude of the warming imposed (Donelson et al., 2016). Also for contaminants, there is mixed evidence in invertebrates: some studies showed parental exposure increasing the tolerance of the offspring (e.g., Brausch & Salice, 2011; Kim et al., 2014; Reátegui‐Zirena et al., 2017), while other studies showed the opposite (Pölkki et al., 2012; Schultz et al., 2016; Yu et al., 2016).

Parental pesticide exposure resulted in slightly larger offspring (ca. 3%) when the offspring were exposed to the pesticide compared to exposed offspring whose parents had not been exposed to the pesticide. This may suggest the occurrence of transgenerational acclimation to pesticide exposure (see Kim, Lee, Yu, & Kim, 2012; but see Brausch & Salice, 2011). Yet, this could also be the result of the combined survival selection imposed by CPF during the parental and the offspring generations, thereby selecting only the fittest larvae, in combination with overcompensatory feeding in offspring exposed to the pesticide whereby animals aimed to reduce the energetic losses due to toxicant effects (e.g., detoxification or damage repair). Overcompensatory feeding has been shown to result in increased body size (Jager, Barsi, & Ducrot, 2013).

#### 4.2.3 (Q3) Does parental exposure to warming and/or to a pesticide shape the synergism between warming and pesticide exposure in the offspring?

A key finding was that the widespread synergism between warming and pesticide exposure (Liess et al., 2016; Noyes & Lema, 2015; Noyes et al., 2009) disappeared in offspring whose parents had been exposed to the pesticide under warming. This was because the joint exposure of the parents to both warming and the pesticide made the offspring more vulnerable to the pesticide. Indeed, the effect of the pesticide on survival was much stronger at 20°C in the offspring of the parents who had been exposed to both warming and the pesticide, compared to offspring whose parents had been exposed to neither, or only one of the stressors. Likely, being exposed to both stressors (CPF at the high temperature) resulted in parents of a lower quality which in turn negatively affected the offspring quality. This is the first demonstration that transgenerational effects may determine how stressors will interact in the offspring generation.

While the synergetic effect between warming and pesticide exposure on survival was apparent in both the parental and the offspring generation, this synergism was absent in terms of size and development time. Possibly, the synergistic effect on survival had removed the weakest larvae.

## 5 CONCLUSIONS

A first key finding was that offspring from stressed parents (exposed to warming and/or pesticide exposure) had reduced survival and were not better at dealing with the same stressors. The associated reassuring take‐home message is that parental exposure to warming and/or pesticide does not buffer the offspring of this vector species against these global change stressors. Our findings contrast with the increasing number of studies indicating that transgenerational effects have the potential to buffer the effect of rapid environmental change on the offspring (Donelson et al., 2018; Munday, 2014). Parental exposure to warming is even being considered as a strategy to enhance species resilience to warming (Chakravarti et al., 2016). Yet, our results are consistent with the general pattern for transgenerational effects not being beneficial for the offspring (Uller, Nakagawa, & English, 2013). While adaptive transgenerational plasticity can facilitate population persistence until long‐term genetic adaptation and may even accelerate adaptive evolution (Diamond & Martin, 2016; Ghalambor et al., 2007), the here observed plasticity is not only maladaptive for the offspring generation but likely also makes it for the mosquito populations more difficult to develop resistance to warming and pollution (but see Stoks et al., 2016). This may suggest that under global warming, CPF‐based pest control of this vector species may become more efficient. The epidemiological implications are less easy to predict and need, amongst others, consideration of how besides life history also the immune competence and vector capacity of the mosquitoes changes and how the viruses and pathogens themselves respond to warming (Kilpatrick et al., 2008; Paz, 2015). Moreover, while our results are an important step to predict climate change effects on vector life‐history traits, future studies should also take into account the effects of daily temperature variation which may also affect vector competence (Parham et al., 2015).

A second key finding was that the widespread synergism between warming and pollutants (Moe et al., 2013; Noyes et al., 2009) was detected in both generations, yet disappeared in the offspring of parents exposed to both stressors because this made the pesticide more toxic even in the absence of warming. The associated take‐home message is that transgenerational effects may critically modify the presence of synergisms which may explain why this key synergism at the interface of ecotoxicology and global change biology is not always detected (e.g., Scheil & Köhler, 2009; Talent, 2005). This finding is important for understanding effects of global warming as by 2100 larger temperature fluctuations are to be expected (IPCC 2013; Wang & Dillon, 2014), which may lead to counterintuitive situations where offspring are sometimes exposed to lower temperatures than their parents.

Together, our results underscore the importance of considering transgenerational plasticity not only when assessing the impact of warming (e.g., Donelson et al., 2016; Shama et al., 2014; Veilleux et al., 2015) and of pollutants (e.g., Costa et al., 2014; Pölkki et al., 2012; Schultz et al., 2016), but also when considering the impact of pollutants in a warming world. Our results thereby highlight the importance of integrating the emerging views of multistressor studies (Liess et al., 2016; Stoks, Geerts, & De Meester, 2014) and studies on transgenerational plasticity (Donelson et al., 2016; Guillaume et al., 2016) to understand the fate of species under global change.

## CONFLICT OF INTEREST

None declared.

## DATA ARCHIVING STATEMENT

Data available from the Dryad Digital Repository: https://doi.org/10.5061/dryad.3fp5c

## References

[eva12605-bib-0001] Atkinson, D. (1994). Temperature and organism size—A biological law for ectotherms? Advances in Ecological Research, 25, 1–58. https://doi.org/10.1016/s0065-2504(08)60212-3

[eva12605-bib-0002] Bates, D. , Maechler, M. , Bolker, B. , & Walker, S. (2015). Fitting linear mixed‐effects models using lme4. Journal of Statistical Software, 67, 1–48.

[eva12605-bib-0003] Bhandari, R. K. , vom Saal, F. S. , & Tillitt, D. E. (2015). Transgenerational effects from early developmental exposures to bisphenol A or 17alpha‐ethinylestradiol in medaka, *Oryzias latipes* . Scientific Reports, 5, 9303 https://doi.org/10.1038/srep09303 25790734 10.1038/srep09303 PMC4366817

[eva12605-bib-0004] Bonduriansky, R. , Crean, A. J. , & Day, T. (2012). The implications of nongenetic inheritance for evolution in changing environments. Evolutionary Applications, 5, 192–201. https://doi.org/10.1111/j.1752-4571.2011.00213.x 25568041 10.1111/j.1752-4571.2011.00213.x PMC3353344

[eva12605-bib-0005] Bonduriansky, R. , & Day, T. (2009). Nongenetic inheritance and its evolutionary implications. Annual Review of Ecology, Evolution, and Systematics, 40, 103–125. https://doi.org/10.1146/annurev.ecolsys.39.110707.173441

[eva12605-bib-0006] Bonduriansky, R. , & Head, M. (2007). Maternal and paternal condition effects on offspring phenotype in *Telostylinus angusticollis* (Diptera: Neriidae). Journal of Evolutionary Biology, 20, 2379–2388. https://doi.org/10.1111/j.1420-9101.2007.01419.x 17956399 10.1111/j.1420-9101.2007.01419.x

[eva12605-bib-0007] Brausch, J. M. , & Salice, C. J. (2011). Effects of an environmentally realistic pesticide mixture on *Daphnia magna* exposed for two generations. Archives of Environmental Contamination and Toxicology, 61, 272–279. https://doi.org/10.1007/s00244-010-9617-z 21069312 10.1007/s00244-010-9617-z

[eva12605-bib-0008] Buchwalter, D. B. , Jenkins, J. J. , & Curtis, L. R. (2002). Respiratory strategy is a major determinant of [^3^H]water and [^14^C]chlorpyrifos uptake in aquatic insects. Canadian Journal of Fisheries and Aquatic Sciences, 59, 1315–1322. https://doi.org/10.1139/f02-107

[eva12605-bib-0009] Buchwalter, D. B. , Jenkins, J. J. , & Curtis, L. R. (2003). Temperature influences on water permeability and chlorpyrifos uptake in aquatic insects with differing respiratory strategies. Environmental Toxicology and Chemistry, 22, 2806–2812. https://doi.org/10.1897/02-350 14587925 10.1897/02-350

[eva12605-bib-0010] Chakravarti, L. J. , Jarrold, M. D. , Gibbin, E. M. , Christen, F. , Massamba‐N'Siala, G. , Blier, P. U. , & Calosi, P. (2016). Can trans‐generational experiments be used to enhance species resilience to ocean warming and acidification? Evolutionary Applications, 9, 1133–1146. https://doi.org/10.1111/eva.12391 27695521 10.1111/eva.12391 PMC5039326

[eva12605-bib-0011] Ciota, A. T. , Matacchiero, A. C. , Kilpatrick, A. M. , & Kramer, L. D. (2014). The effect of temperature on life history traits of *Culex* mosquitoes. Journal of Medical Entomology, 51, 55–62. https://doi.org/10.1603/ME13003 24605453 10.1603/me13003 PMC3955846

[eva12605-bib-0012] Corrales, J. , Thornton, C. , White, M. , & Willett, K. L. (2014). Multigenerational effects of benzo [a] pyrene exposure on survival and developmental deformities in zebrafish larvae. Aquatic Toxicology, 148, 16–26. https://doi.org/10.1016/j.aquatox.2013.12.028 24440964 10.1016/j.aquatox.2013.12.028 PMC3940271

[eva12605-bib-0013] Costa, M. A. , Moscardini, V. F. , da Costa Gontijo, P. , Carvalho, G. A. , de Oliveira, R. L. , & de Oliveira, H. N. (2014). Sublethal and transgenerational effects of insecticides in developing *Trichogramma galloi* (Hymenoptera: Trichogrammatidae). Ecotoxicology, 23, 1399–1408. https://doi.org/10.1007/s10646-014-1282-y 25011923 10.1007/s10646-014-1282-y

[eva12605-bib-0014] Curley, J. P. , Mashoodh, R. , & Champagne, F. A. (2011). Epigenetics and the origins of paternal effects. Hormones and Behavior, 59, 306–314. https://doi.org/10.1016/j.yhbeh.2010.06.018 20620140 10.1016/j.yhbeh.2010.06.018 PMC2975825

[eva12605-bib-0015] Diamond, S. E. , & Martin, R. A. (2016). The interplay between plasticity and evolution in response to human‐induced environmental change. F1000Research, 5, 2835 https://doi.org/10.12688/f1000research 28003883 10.12688/f1000research.9731.1 PMC5147521

[eva12605-bib-0016] Donelson, J. M. , Salinas, S. , Munday, P. L. , & Shama, L. N. S. (2018). Transgenerational plasticity and climate change experiments: Where do we go from here? Global Change Biology, 24, 13–34. 29024256 10.1111/gcb.13903

[eva12605-bib-0017] Donelson, J. M. , Wong, M. , Booth, D. J. , & Munday, P. L. (2016). Transgenerational plasticity of reproduction depends on rate of warming across generations. Evolutionary Applications, 9, 1072–1081. https://doi.org/10.1111/eva.12386 27695516 10.1111/eva.12386 PMC5039321

[eva12605-bib-0018] Eaton, D. L. , Daroff, R. B. , Autrup, H. , Bridges, J. , Buffler, P. , Costa, L. G. , … Spencer, P. S. (2008). Review of the toxicology of chlorpyrifos with an emphasis on human exposure and neurodevelopment. Critical Reviews in Toxicology, 38, 1–125. https://doi.org/10.1080/10408440802272158 10.1080/10408440802272158 18726789

[eva12605-bib-0019] Farajollahi, A. , Fonseca, D. M. , Kramer, L. D. , & Kilpatrick, A. M. (2011). “Bird biting” mosquitoes and human disease: A review of the role of *Culex pipiens* complex mosquitoes in epidemiology. Infection, Genetics and Evolution, 11, 1577–1585. https://doi.org/10.1016/j.meegid.2011.08.013 10.1016/j.meegid.2011.08.013 PMC3190018 21875691

[eva12605-bib-0020] Ferrer, A. , Dorn, S. , & Mazzi, D. (2013). Cross‐generational effects of temperature on flight performance, and associated life‐history traits in an insect. Journal of Evolutionary Biology, 26, 2321–2330. https://doi.org/10.1111/jeb.12218 23981249 10.1111/jeb.12218

[eva12605-bib-0021] Fonseca, D. M. , Keyghobadi, N. , Malcolm, C. A. , Mehmet, C. , Schaffner, F. , Mogi, M. , … Wilkerson, R. C. (2004). Emerging vectors in the *Culex pipiens* complex. Science (New York), 303, 1535–1538. https://doi.org/10.1126/science.1094247 10.1126/science.1094247 15001783

[eva12605-bib-0022] Fox, J. (2003). Effect displays in R for generalised linear models. Journal of Statistical Software, 8, 1–27.

[eva12605-bib-0023] Fox, J. , & Weisberg, S. (2011). An R companion to applied regression (2nd ed.). Thousand Oaks, CA: Sage.

[eva12605-bib-0024] Ghalambor, C. K. , McKay, J. K. , Carroll, S. P. , & Reznick, D. N. (2007). Adaptive versus non‐adaptive phenotypic plasticity and the potential for contemporary adaptation in new environments. Functional Ecology, 21, 394–407. https://doi.org/10.1111/j.1365-2435.2007.01283.x

[eva12605-bib-0025] Guillaume, A. S. , Monro, K. , & Marshall, D. J. (2016). Transgenerational plasticity and environmental stress: Do paternal effects act as a conduit or a buffer? Functional Ecology, 30, 1175–1184. https://doi.org/10.1111/1365-2435.12604

[eva12605-bib-0026] Hahn, T. , Schenk, K. , & Schulz, R. (2002). Environmental chemicals with known endocrine potential affect yolk protein content in the aquatic insect *Chironomus riparius* . Environmental Pollution, 120, 525–528. https://doi.org/10.1016/S0269-7491(02)00189-6 12442778 10.1016/s0269-7491(02)00189-6

[eva12605-bib-0027] Hariprasad, T. P. N. , & Shetty, N. J. (2017). Sublethal and transgenerational effects of alphamethrin on life history traits of *Anopheles stephensi* (Diptera: Culicidae), a malaria mosquito. Canadian Entomologist, 149, 251–264. https://doi.org/10.4039/tce.2016.57

[eva12605-bib-0028] Hemingway, J. , Field, L. , & Vontas, J. (2002). An overview of insecticide resistance. Science, 298, 96–97. https://doi.org/10.1126/science.1078052 12364782 10.1126/science.1078052

[eva12605-bib-0029] Ho, D. H. , & Burggren, W. W. (2010). Epigenetics and transgenerational transfer: A physiological perspective. The Journal of Experimental Biology, 213, 3–16. https://doi.org/10.1242/jeb.019752 20008356 10.1242/jeb.019752

[eva12605-bib-0030] Holmstrup, M. , Bindesbøl, A.‐M. , Oostingh, G. J. , Duschl, A. , Scheil, V. , Köhler, H.‐R. , … Spurgeon, D. J. (2010). Interactions between effects of environmental chemicals and natural stressors: A review. Science of the Total Environment, 408, 3746–3762. https://doi.org/10.1016/j.scitotenv.2009.10.067 19922980 10.1016/j.scitotenv.2009.10.067

[eva12605-bib-0031] Huestis, D. L. , Yaro, A. S. , Traoré, A. I. , Adamou, A. , Kassogué, Y. , Diallo, M. , … Lehmann, T. (2011). Variation in metabolic rate of *Anopheles gambiae* and *A. arabiensis* in a Sahelian village. Journal of Experimental Biology, 214, 2345–2353. https://doi.org/10.1242/jeb.054668 21697426 10.1242/jeb.054668 PMC3120220

[eva12605-bib-0032] IPCC (2013). Climate change 2013: The physical science basis. Contribution of working group i to the fifth assessment report of the intergovernmental panel on climate change (p. 1535). Cambridge, UK and New York, NY: Cambridge University Press.

[eva12605-bib-0033] Jager, T. , Barsi, A. , & Ducrot, V. (2013). Hormesis on life‐history traits: Is there such thing as a free lunch? Ecotoxicology, 22, 263–270. https://doi.org/10.1007/s10646-012-1022-0 23179410 10.1007/s10646-012-1022-0

[eva12605-bib-0034] Jensen, N. , Allen, R. M. , & Marshall, D. J. (2014). Adaptive maternal and paternal effects: Gamete plasticity in response to parental stress. Functional Ecology, 28, 724–733. https://doi.org/10.1111/1365-2435.12195

[eva12605-bib-0035] Kielland, Ø. N. , Bech, C. , & Einum, S. (2017). No evidence for thermal transgenerational plasticity in metabolism when minimizing the potential for confounding effects. Proceedings of the Royal Society B: Biological Sciences, 284, 20162494 https://doi.org/10.1098/rspb.2016.2494 28077777 10.1098/rspb.2016.2494 PMC5247506

[eva12605-bib-0501] Kilpatrick, A. M. (2011). Review globalization, land use, and the invasion of West Nile Virus. Science, 334, 323–328. 22021850 10.1126/science.1201010 PMC3346291

[eva12605-bib-0036] Kilpatrick, A. M. , Meola, M. A. , Moudy, R. M. , & Kramer, L. D. (2008). Temperature, viral genetics, and the transmission of West Nile virus by *Culex pipiens* Mosquitoes. Plos Pathogens, 4, e1000092 https://doi.org/10.1371/journal.ppat.1000092 18584026 10.1371/journal.ppat.1000092 PMC2430533

[eva12605-bib-0037] Kim, H. Y. , Lee, M. J. , Yu, S. H. , & Kim, S. D. (2012). The individual and population effects of tetracycline on *Daphnia magna* in multigenerational exposure. Ecotoxicology, 21, 993–1002. https://doi.org/10.1007/s10646-012-0853-z 22252291 10.1007/s10646-012-0853-z

[eva12605-bib-0038] Kim, H. Y. , Yu, S. , Jeong, T. Y. , & Kim, S. D. (2014). Relationship between trans‐generational effects of tetracycline on *Daphnia magna* at the physiological and whole organism level. Environmental Pollution, 191, 111–118. https://doi.org/10.1016/j.envpol.2014.04.022 24832921 10.1016/j.envpol.2014.04.022

[eva12605-bib-0039] Kimberly, D. A. , & Salice, C. J. (2015). Multigenerational contaminant exposures produce non‐monotonic, transgenerational responses in *Daphnia magna* . Environmental Pollution, 207, 176–182. https://doi.org/10.1016/j.envpol.2015.09.020 26378969 10.1016/j.envpol.2015.09.020

[eva12605-bib-0040] Koella, J. C. , Saddler, A. , & Karacs, T. P. (2012). Blocking the evolution of insecticide‐resistant malaria vectors with a microsporidian. Evolutionary Applications, 5, 283–292. https://doi.org/10.1111/j.1752-4571.2011.00219.x 25568048 10.1111/j.1752-4571.2011.00219.x PMC3353349

[eva12605-bib-0041] Lenth, R. V. (2016). Least‐squares means: The R package lsmeans. Journal of Statistical Software, 69, 1–33.

[eva12605-bib-0042] Liess, M. , Foit, K. , Knillmann, S. , Schäfer, R. B. , & Liess, H.‐D. (2016). Predicting the synergy of multiple stress effects. Scientific Reports, 6, 32965 https://doi.org/10.1038/srep32965 27609131 10.1038/srep32965 PMC5017025

[eva12605-bib-0043] Liu, N. (2015). Insecticide resistance in mosquitoes: Impact, mechanisms, and research directions. Annual Review of Entomology, 60, 537–559. https://doi.org/10.1146/annurev-ento-010814-020828 10.1146/annurev-ento-010814-020828 25564745

[eva12605-bib-0044] Lydy, M. J. , Belden, J. B. , & Ternes, M. A. (1999). Effects of temperature on the toxicity of m‐parathion, chlorpyrifos, and pentachlorobenzene to *Chironomus tentans* . Archives of Environmental Contamination and Toxicology, 37, 542–547. https://doi.org/10.1007/s002449900550 10508903 10.1007/s002449900550

[eva12605-bib-0045] Mazanti, L. , Rice, C. , Bialek, K. , Sparling, D. , Stevenson, C. , Johnson, W. E. , … Rheinstein, J. (2003). Aqueous‐phase disappearance of atrazine, metolachlor, and chlorpyrifos in laboratory aquaria and outdoor macrocosms. Archives of Environmental Contamination and Toxicology, 44, 67–76. https://doi.org/10.1007/s00244-002-1259-3 12434220 10.1007/s00244-002-1259-3

[eva12605-bib-0046] Moe, S. J. , De Schamphelaere, K. , Clements, W. H. , Sorensen, M. T. , Van den Brink, P. J. , & Liess, M. (2013). Combined and interactive effects of global climate change and toxicants on populations and communities. Environmental Toxicology and Chemistry, 32, 49–61. https://doi.org/10.1002/etc.2045 23147390 10.1002/etc.2045 PMC3601420

[eva12605-bib-0047] Munday, P. L. (2014). Transgenerational acclimation of fishes to climate change and ocean acidification. F1000Prime Reports, 6, 99. 25580253 10.12703/P6-99 PMC4229724

[eva12605-bib-0048] Noyes, P. D. , & Lema, S. C. (2015). Forecasting the impacts of chemical pollution and climate change interactions on the health of wildlife. Current Zoology, 61, 669–689. https://doi.org/10.1093/czoolo/61.4.669

[eva12605-bib-0049] Noyes, P. D. , McElwee, M. K. , Miller, H. D. , Clark, B. W. , Van Tiem, L. A. , Walcott, K. C. , … Levin, E. D. (2009). The toxicology of climate change: Environmental contaminants in a warming world. Environment International, 35, 971–986. https://doi.org/10.1016/j.envint.2009.02.006 19375165 10.1016/j.envint.2009.02.006

[eva12605-bib-0050] Op de Beeck, L. , Verheyen, J. , Olsen, K. , & Stoks, R. (2017). Negative effects of pesticides under global warming can be counteracted by a higher degradation rate and thermal adaptation. Journal of Applied Ecology, 54, 1847–1855. https://doi.org/10.1111/1365-2664.12919

[eva12605-bib-0051] Parham, P. E. , Waldock, J. , Christophides, G. K. , Hemming, D. , Agusto, F. , Evans, K. J. , … Michael, E. (2015). Climate, environmental and socio‐economic change: Weighing up the balance in vector‐borne disease transmission. Philosophical Transactions of the Royal Society B: Biological Sciences, 370, 20130551 https://doi.org/10.1098/rstb.2013.0551 10.1098/rstb.2013.0551 PMC4342957 25688012

[eva12605-bib-0052] Paz, S. (2015). Climate change impacts on West Nile virus transmission in a global context. Philosophical Transactions of the Royal Society B: Biological Sciences, 370, 20130561 https://doi.org/10.1098/rstb.2013.0561 10.1098/rstb.2013.0561 PMC4342965 25688020

[eva12605-bib-0053] Pölkki, M. , Kangassalo, K. , & Rantala, M. J. (2012). Transgenerational effects of heavy metal pollution on immune defense of the blow fly *Protophormia terraenovae* . PLoS One, 7, e38832 https://doi.org/10.1371/journal.pone.0038832 22719959 10.1371/journal.pone.0038832 PMC3373569

[eva12605-bib-0054] Prud'homme, S. M. , Chaumot, A. , Cassar, E. , David, J. P. , & Reynaud, S. (2017). Impact of micropollutants on the life‐history traits of the mosquito *Aedes aegypti*: On the relevance of transgenerational studies. Environmental Pollution, 220, 242–254. https://doi.org/10.1016/j.envpol.2016.09.056 27667679 10.1016/j.envpol.2016.09.056

[eva12605-bib-0055] R Development Core Team (2017). R: A language and environment for statistical computing. Vienna, Austria: R Foundation for Statistical Computing Retrieved from http://www.R-project.org/

[eva12605-bib-0056] Reátegui‐Zirena, E. G. , Fidder, B. N. , Olson, A. D. , Dawson, D. E. , Bilbo, T. R. , & Salice, C. J. (2017). Transgenerational endpoints provide increased sensitivity and insight into multigenerational responses of *Lymnaea stagnalis* exposed to cadmium. Environmental Pollution, 224, 572–580. https://doi.org/10.1016/j.envpol.2017.02.040 28274592 10.1016/j.envpol.2017.02.040

[eva12605-bib-0057] Rubach, M. N. , Baird, D. J. , Boerwinkel, M. C. , Maund, S. J. , Roessink, I. , & Van den Brink, P. J. (2012). Species traits as predictors for intrinsic sensitivity of aquatic invertebrates to the insecticide chlorpyrifos. Ecotoxicology, 21, 2088–2101. https://doi.org/10.1007/s10646-012-0962-8 22711550 10.1007/s10646-012-0962-8 PMC3431471

[eva12605-bib-0058] Salinas, S. , & Munch, S. B. (2012). Thermal legacies: Transgenerational effects of temperature on growth in a vertebrate. Ecology Letters, 15, 159–163. https://doi.org/10.1111/j.1461-0248.2011.01721.x 22188553 10.1111/j.1461-0248.2011.01721.x

[eva12605-bib-0059] Scheil, V. , & Köhler, H.‐R. (2009). Influence of nickel chloride, chlorpyrifos, and imidacloprid in combination with different temperatures on the embryogenesis of the zebrafish *Danio rerio* . Archives of Environmental Contamination and Toxicology, 56, 238–243. https://doi.org/10.1007/s00244-008-9192-8 18661094 10.1007/s00244-008-9192-8

[eva12605-bib-0060] Schultz, C. L. , Wamucho, A. , Tsyusko, O. V. , Unrine, J. M. , Crossley, A. , Svendsen, C. , & Spurgeon, D. J. (2016). Multigenerational exposure to silver ions and silver nanoparticles reveals heightened sensitivity and epigenetic memory in *Caenorhabditis elegans* . Proceedings of the Royal Society B: Biological Sciences, 283, 20152911 https://doi.org/10.1098/rspb.2015.2911 27306046 10.1098/rspb.2015.2911 PMC4920304

[eva12605-bib-0061] Shama, L. N. S. , Strobel, A. , Mark, F. C. , & Wegner, K. M. (2014). Transgenerational plasticity in marine sticklebacks: Maternal effects mediate impacts of a warming ocean. Functional Ecology, 28, 1482–1493. https://doi.org/10.1111/1365-2435.12280

[eva12605-bib-0062] Shama, L. N. S. , & Wegner, K. M. (2014). Grandparental effects in marine sticklebacks: Transgenerational plasticity across multiple generations. Journal of Evolutionary Biology, 27, 2297–2307. https://doi.org/10.1111/jeb.12490 25264208 10.1111/jeb.12490

[eva12605-bib-0063] Stoks, R. , Geerts, A. N. , & De Meester, L. (2014). Evolutionary and plastic responses of freshwater invertebrates to climate change: Realized patterns and future potential. Evolutionary Applications, 7, 42–55. https://doi.org/10.1111/eva.12108 24454547 10.1111/eva.12108 PMC3894897

[eva12605-bib-0064] Stoks, R. , Govaert, L. , Pauwels, K. , Jansen, B. , & De Meester, L. (2016). Resurrecting complexity: The interplay of plasticity and rapid evolution in the multiple trait response to strong changes in predation pressure in the water flea *Daphnia magna* . Ecology Letters, 19, 180–190. https://doi.org/10.1111/ele.12551 10.1111/ele.12551 26647739

[eva12605-bib-0065] Storm, J. J. , & Lima, S. L. (2010). Mothers forewarn offspring about predators: A transgenerational maternal effect on behavior. The American Naturalist, 17, 382–390. https://doi.org/10.1086/650443 10.1086/650443 20109061

[eva12605-bib-0066] Talent, L. G. (2005). Effect of temperature on toxicity of a natural pyrethrin pesticide to green anole lizards (*Anolis carolinensis*). Environmental Toxicology and Chemistry, 24, 3113–3116. https://doi.org/10.1897/05-053R.1 16445093 10.1897/05-053r.1

[eva12605-bib-0067] Tran, T. T. , Janssens, L. , Dinh, K. V. , Op de Beeck, L. , & Stoks, R. (2016). Evolution determines how global warming and pesticide exposure will shape predator‐prey interactions with vector mosquitoes. Evolutionary Applications, 9, 818–830. https://doi.org/10.1111/eva.12390 27330557 10.1111/eva.12390 PMC4908467

[eva12605-bib-0068] Uller, T. , Nakagawa, S. , & English, S. (2013). Weak evidence for anticipatory parental effects in plants and animals. Journal of Evolutionary Biology, 26, 2161–2170. https://doi.org/10.1111/jeb.12212 23937440 10.1111/jeb.12212

[eva12605-bib-0069] Van Dinh, K. , Janssens, L. , Debecker, S. , & Stoks, R. (2014). Warming increases chlorpyrifos effects on predator but not anti‐predator behaviours. Aquatic Toxicology, 152, 215–221. https://doi.org/10.1016/j.aquatox.2014.04.011 24792152 10.1016/j.aquatox.2014.04.011

[eva12605-bib-0070] Veilleux, H. D. , Ryu, T. , Donelson, J. M. , Van Herwerden, L. , Seridi, L. , Ghosheh, Y. , … Munday, P. L. (2015). Molecular processes of transgenerational acclimation to a warming ocean. Nature Climate Change, 5, 1074–1078. https://doi.org/10.1038/nclimate2724

[eva12605-bib-0071] Walsh, M. R. , Whittington, D. , & Funkhouser, C. (2014). Thermal transgenerational plasticity in natural populations of *Daphnia* . Integrative and Comparative Biology, 54, 822–829. https://doi.org/10.1093/icb/icu078 24948139 10.1093/icb/icu078

[eva12605-bib-0072] Wang, G. , & Dillon, M. E. (2014). Recent geographic convergence in diurnal and annual temperature cycling flattens global thermal profiles. Nature Climate Change, 4, 988–992. https://doi.org/10.1038/nclimate2378

[eva12605-bib-0073] WHO (2017). WHOPES‐recommended compounds and formulations for control of mosquito larvae. Retrieved from http://www.paho.org/hq/index.php?option=com_docman&task=doc_details&gid=18314&Itemid=2518&lang=en

[eva12605-bib-0074] WHOPES (2005). Guidelines for laboratory and field testing of mosquito larvicides (pp. 1–41). Geneva, Switzerland: World Health Organization.

[eva12605-bib-0075] Yu, C. W. , & Liao, V. H. (2016). Transgenerational reproductive effects of arsenite are associated with H3K4 dimethylation and SPR‐5 downregulation in *Caenorhabditis elegans* . Environmental Science and Technology, 50, 10673–10681. https://doi.org/10.1021/acs.est.6b02173 27579588 10.1021/acs.est.6b02173

[eva12605-bib-0076] Yu, Z. , Zhang, J. , & Yin, D. (2016). Multigenerational effects of heavy metals on feeding, growth, initial reproduction and antioxidants in *Caenorhabditis elegans* . PLoS One, 11, e0154529 https://doi.org/10.1371/journal.pone.0154529 27116222 10.1371/journal.pone.0154529 PMC4846010

