# Supplementary material for: Transgenerational interactions between pesticide exposure and warming in a vector mosquito
Source: Evol Appl. 2018 Mar 5;11(6):906–17. doi: 10.1111/eva.12605 (PMC5999214; doi:10.1111/eva.12605)
Supplement: Supplementary file 1 [file EVA-11-906-s001.docx]

**Appendix 1: Detailed experimental procedure**

Parental generation (F0)

We started the experiment in the parental (F0) generation with 27 rafts for each of the 4 temperature-by-pesticide treatment combinations, giving a total of 108 egg rafts (see Step 1, Figure S1 for the experimental scheme). Note that at this part of the experiment only the temperature treatment (Temperature F0) had started. Each raft was individually hatched in a 200 mL glass vial filled with 125 mL of dechlorinated tap water at 20°C or 24°C. Three days after hatching, when the majority of the larvae were in the second instar (both at 20°C and 24°C), 40 larvae hatched from a single raft were reared together in an initial vial (step 2). Each initial vial only contained larvae that hatched from a single, unique egg raft. At each temperature, we had 9 sets of 6 initial vials. Later on in the experiment vials were pooled and larvae were redistributed, but always within the same set to obtain exposure vials that underwent the pesticide treatment (step 3).

When larvae reached the final larval stage (L4), we started the pesticide treatment (Pesticide F0). At that moment, larvae from a subset of 3 initial vials of the same set at a given temperature (20°C or 24°C) were pooled and redistributed among two exposure vials of 30 larvae (one control vial and one vial with CPF) (steps 3&4). For each initial set of 6 initial vials this resulted in 2 control exposure vials and 2 CPF exposure vials. This resulted per temperature in 36 exposure vials (18 replicate exposure vials per treatment combination) for larval survival during the 5 day exposure period (see step 4).

At metamorphosis the two control vials (two pesticide vials) at the same temperature, which were created from the same set of vials, were pooled in one control insectary (pesticide insectary) (step 5). This resulted per temperature in 18 insectaries (9 insectaries per treatment combination). As we pooled animals from two control (CPF) exposure vials in one control (CPF) insectary, we were no longer able to identify the vial ID of the adults in the insectary. Therefore, we had 9 replicate insectaries per temperature-by-pesticide treatment combination for survival until metamorphosis, development time and body size.

In summary, at each temperature we started with 9 sets of six vials consisting of 54 egg rafts and installed 36 exposure vials (18 control vials and 18 CPF vials), 18 insectaries (9 insectaries per treatment combination).

Offspring generation (F1)

To start the offspring (F) generation, from each insectary in the parental generation 12 vials were started, a set of six vials at 20°C and a set of six vials at 24°C (step 6). In total, we used 432 egg rafts from 36 parental insectaries to start the offspring generation. In the offspring generation we followed the same pooling and redistribution steps as in the parental generation (steps 7-11). This resulted in the same number of exposure vials (18) and insectaries (9) per offspring treatment combination as observed in the parental generation. Note there are now 16 treatment combinations: 2 parental warming x 2 parental pesticide exposure x 2 offspring warming x 2 offspring pesticide exposure.

Example

To further illustrate and clarify the experimental procedure we here focus on a specific example trajectory: the vials and larvae of parental set 1 at 20°C . At the start, parental set 1 at 20°C included 6 initial vials (two subsets of 3 initial vials: subsets 1A and 1B) (see steps 1&2). When larvae reached L4, the three initial vials in each subset were pooled and redistributed among two exposure vials, one control and one pesticide vial (steps 3&4) . For example, we pooled larvae from subset 1A and installed one control exposure vial (called C1) and one CPF exposure vial (called P1). Similarly, from subset 1B we installed one control exposure vial C2 and one CPF exposure vial P2. Once the pupation started, the pupae from exposure vials C1 and C2 (these vials came from the same set 1) were pooled into one insectary called C’1 (step 5). Pupae from exposure vials P1 and P2 were pooled into insectary P’1 (step 5). In the offspring generation, egg rafts from insectary C’1 were used to establish two sets (set 1 at 20°C and set 19 at 24°C, see step 6). From set 1 consisting of 2 subsets (subset 1a and subset1b), we installed two control exposure vials (c1 and c2) and two CPF exposure vials (p1 and p2) at 20°C (see step 10). As in the parental generation, insectary c’1 (p’1) was established from the animals emerging from vials c1 and c2 (p1 and p2) (see step 11).


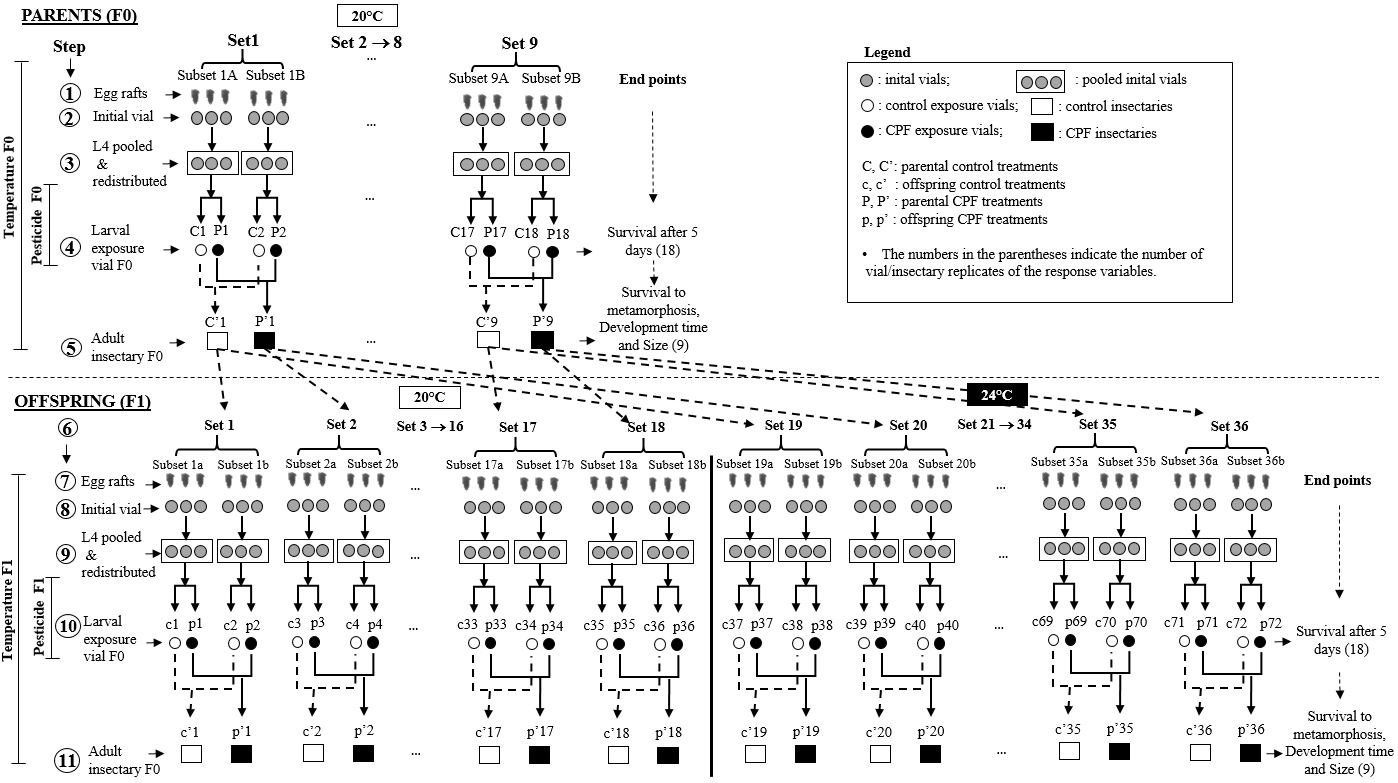


Figure S1: Detailed experimental procedure. The figure only presents the half of the parental treatment combination at 20°C. The half of the parental generation at 24°C is similar to this design.
