# Supplementary material for: Transgenerational interactions between pesticide exposure and warming in a vector mosquito
Source: Evol Appl. 2018 Mar 5;11(6):906–17. doi: 10.1111/eva.12605 (PMC5999214; doi:10.1111/eva.12605)
Supplement: Supplementary file 2 [file EVA-11-906-s002.docx]

**Appendix 2. Larval survival during the exposure period, development time and sex ratio at metamorphosis**

**Data analysis for effects on larval survival during the five day pesticide exposure period**

Survival was scored as 1 (alive) and 0 (dead) for each larva and was analyzed using a generalized linear mixed model with a binomial error structure and the logit link function. To take into account the experimental procedure, we added specific random factors to the model. In the first generation, vial-F0 (the exposure vial of a larva in the F0 generation) nested in subset-F0, subset-F0 nested in Set-F0 and set-F0 were added. In the second generation, vial-F1 nested in subset-F1, subset-F1 nested in set-F1, set-F1 nested in insectary-F0 and insectary-F0 were included.

**Within-generation effects of temperature and pesticide exposure on larval survival during the exposure period in the parental (F0) generation**

In the parental generation, survival of the mosquito larvae during the 5 day exposure period was ca. 100% at 20°C in the solvent control. Survival was negatively affected by warming and especially by pesticide exposure (Table S1, Fig. S2). Moreover, the effect of the pesticide was stronger under warming resulting in a much lower survival at 24°C (77%) than at 20°C (92%) (Temp F0 × Pesticide F0 interaction, Table S1, Fig. S2).

**Within- and transgenerational effects of temperature and pesticide exposure on larval survival and development time in the offspring (F1) generation**

Also in the offspring generation, both warming and especially exposure to the pesticide reduced larval survival (main effects of Temperature F1 and Pesticide F1, Table S2, Fig. S3). As in the first generation, the negative effect of the pesticide was overall stronger under warming. Yet, this was not the case when the parents had been exposed to CPF at 24°C: these offspring already showed a strong CPF-induced reduction in survival at 20°C and no further reduction at 24°C (Temp F0 × Pest F0 × Temp F1 × Pest F1, Table S2, Fig. S3). Overall, parental exposure to warming reduced survival in the offspring (Temp F0, Table S2, Fig. S3).

As in the parental generation, development time was reduced by ca. 28% (ca. 5 days) under warming and by ca. 9% (ca. 1.5 days) under pesticide exposure (main effects Temperature F1 and Pesticide F1, Table 2, Fig. 4C & S4). Metamorphosis was also delayed in response to parental warming (ca. 10%) and parental pesticide exposure (ca. 7%), but only in offspring that was reared at 20°C (Temp F0 × Temp F1, Table 2, Figure 4A; Pest F0 × Temp F1, Table 2, Fig. 4B).

**Within- and transgenerational effects of temperature and pesticide exposure on sex ratio**

In both generations, the sex ratios (# males : # females) were not affected by current warming and pesticide exposure (Fig. S5, Fig. S6). The sex ratio in the offspring were neither influenced by parental warming or pesticide exposure or their interaction with these stressors currently experienced by the offspring (Table S2).

**Table S1.** Effects of temperature and pesticide exposure on larval survival during the exposure period and the sex ratio at metamorphosis of *Culex pipiens* mosquitoes in the parental (F0) generation.

|  |  | Larval survival | | |  | Sex ratio | | |
| --- | --- | --- | --- | --- | --- | --- | --- | --- |
|  |  | df | χ^2^ | *P* |  | df | χ^2^ | *P* |
| Temperature F0 |  | 1 | 13.47 | **<0.001** |  | 1 | 0.061 | 0.805 |
| Pesticide F0 |  | 1 | 35.94 | **<0.001** |  | 1 | 0.031 | 0.860 |
| Temperature F0 × Pesticide F0 |  | 1 | 3.94 | **0.047** |  | 1 | 0.176 | 0.675 |

Significant *P* values (*P* < 0.05) are indicated in bold.

**Table S2.** Effects of temperature and pesticide exposure during the parental (F0) and offspring (F1) generations on larval survival during the exposure period and the sex ratio of *Culex pipiens* mosquitoes in the offspring (F1) generation.

| Effect | Larval survival | | |  | Sex ratio |  |
| --- | --- | --- | --- | --- | --- | --- |
|  | df | χ^2^ | *P* | df | χ^2^ | *P* |
| Temperature F0 (Temp F0) | 1 | 7.65 | **0.006** | 1 | 0.047 | 0.829 |
| Pesticide F0 (Pest F0) | 1 | 0.03 | 0.857 | 1 | 0.008 | 0.930 |
| Temperature F1 (Temp F1) | 1 | 13.74 | **<0.001** | 1 | 0.005 | 0.943 |
| Pesticide F1 (Pest F1) | 1 | 275.94 | **<0.001** | 1 | 0.022 | 0.883 |
| Temp F0 × Pest F0 | 1 | 1.27 | 0.259 | 1 | 0.001 | 0.990 |
| Temp F0 × Temp F1 | 1 | 5.36 | **0.021** | 1 | 0.101 | 0.750 |
| Pest F0 × Temp F1 | 1 | 3.17 | 0.075 | 1 | 0.110 | 0.740 |
| Temp F0 × Pest F1 | 1 | 0.68 | 0.408 | 1 | 0.018 | 0.893 |
| Pest F0 × Pest F1 | 1 | 0.32 | 0.570 | 1 | 0.001 | 0.970 |
| Temp F1 × Pest F1 | 1 | 2.92 | 0.087 | 1 | 0.168 | 0.682 |
| Temp F0 × Pest F0 × Temp F1 | 1 | 0.15 | 0.701 | 1 | 0.182 | 0.670 |
| Temp F0 × Pest F0 × Pest F1 | 1 | 5.52 | **0.019** | 1 | 0.286 | 0.593 |
| Temp F0 × Temp F1 × Pest F1 | 1 | 1.73 | 0.189 | 1 | 0.051 | 0.821 |
| Pest F0 × Temp F1 × Pest F1 | 1 | 3.59 | 0.058 | 1 | 0.044 | 0.834 |
| Temp F0 × Pest F0 × Temp F1 × Pest F1 | 1 | 4.30 | **0.038** | 1 | 0.385 | 0.535 |

Significant *P* values (*P* < 0.05) are indicated in bold.

**
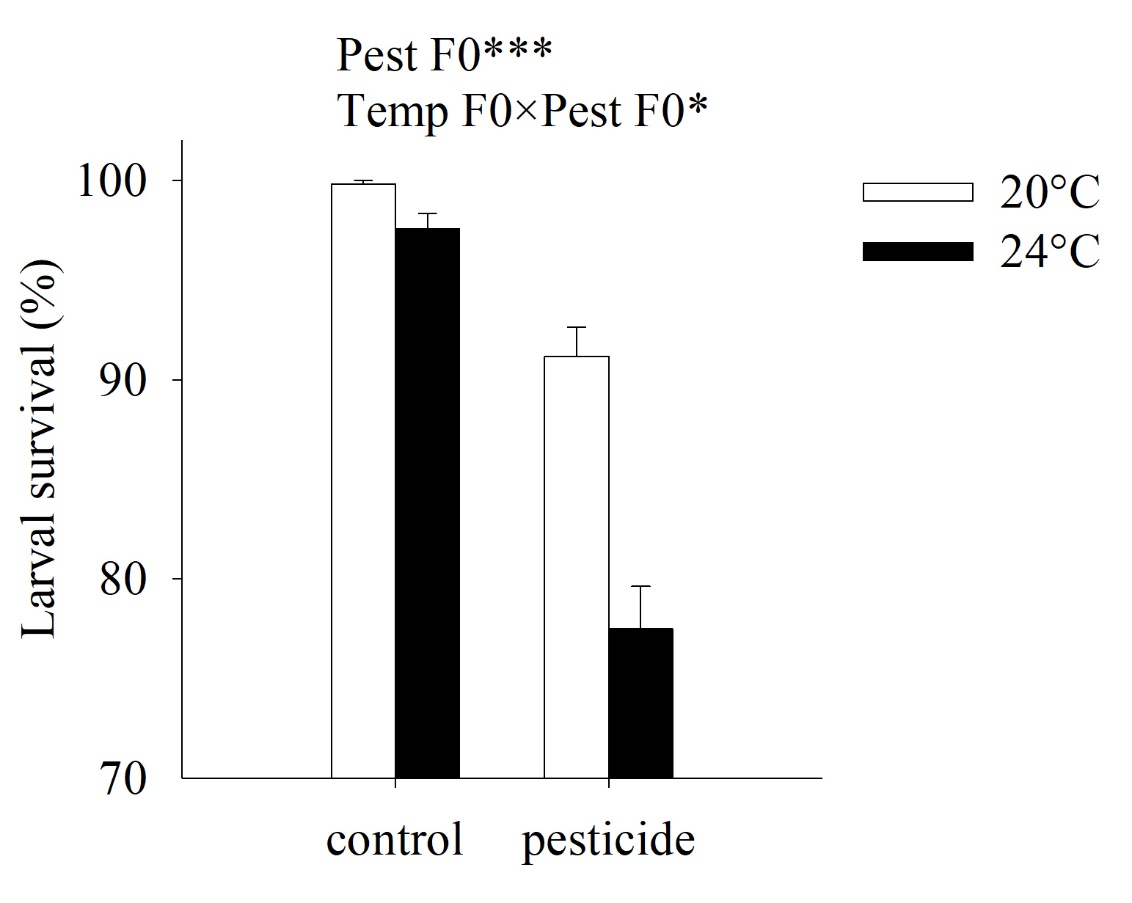
**

Figure S2. Larval survival during the exposure period of *C. pipiens* mosquitoes in the parental generation as a function of temperature and pesticide treatments. Given are LS-means with 1 SE. The asterisks indicate significant effects (* *P* < 0.05, ** *P* < 0.01, *** *P* < 0.001). Sample size: n = 18 for all treatment combinations.

**
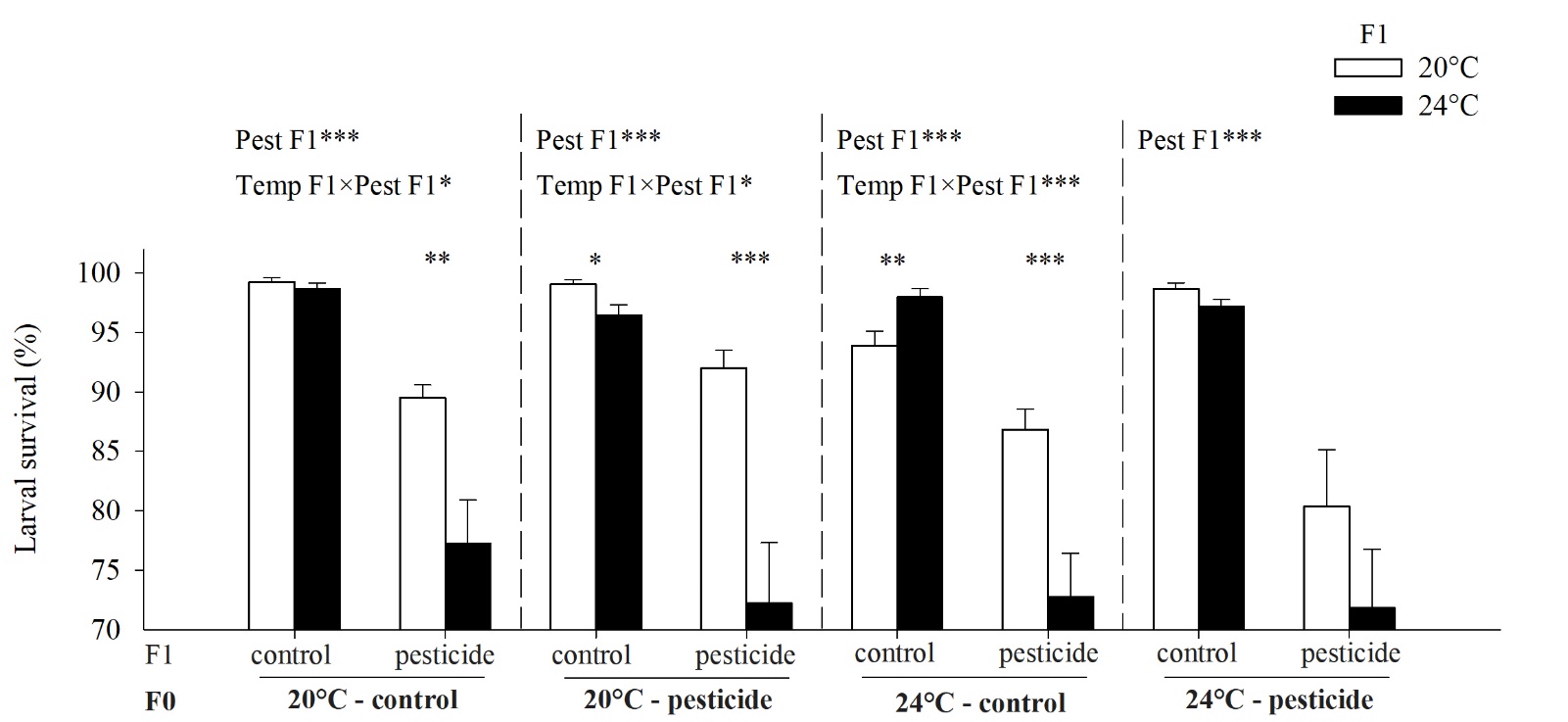
**

Figure S3. Larval survival during the exposure period of *C. pipiens* mosquitoes in the offspring generation as a function of temperature and pesticide treatments in the parental (F0) and offspring (F1) generations. Given are LS-means with 1 SE. The asterisks indicate significant effects of warming for a given pesticide treatment (* *P* < 0.05, ** *P* < 0.01, *** *P* < 0.001). Sample size: n=18 for all treatment combinations.


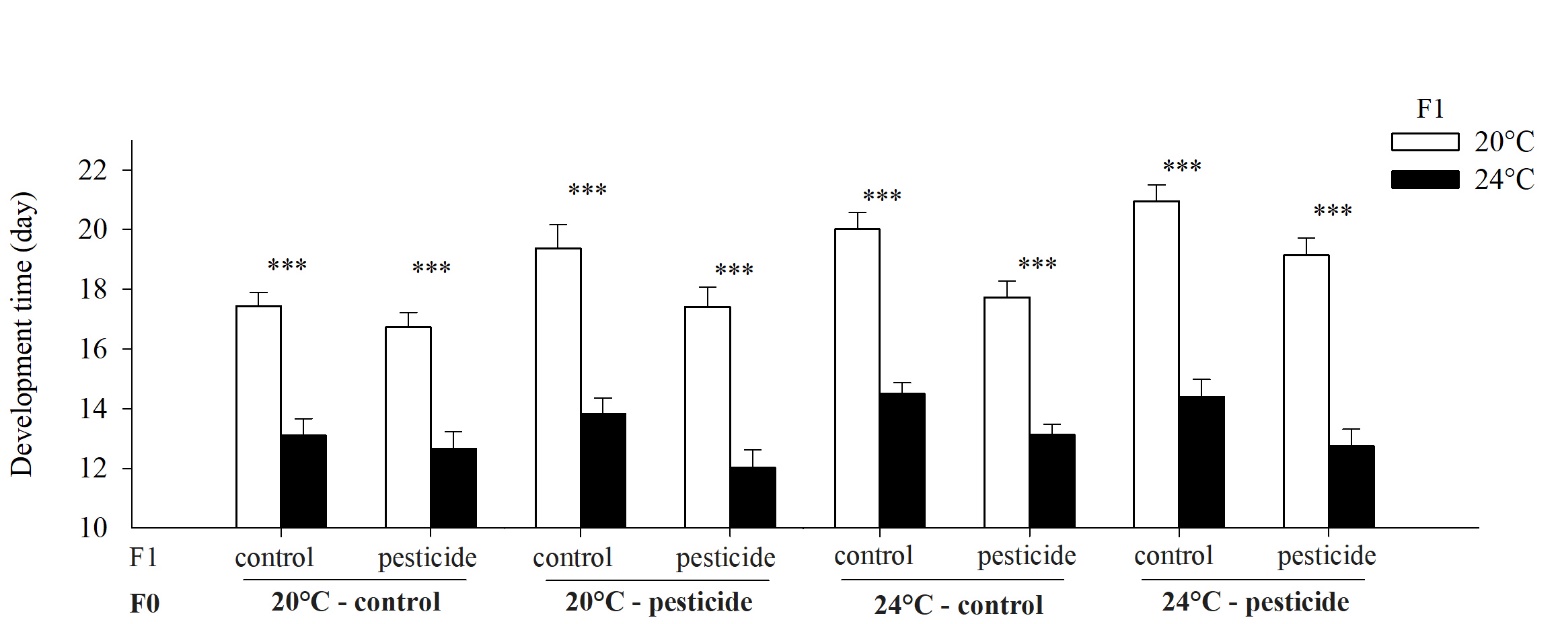
Figure S4. Development time of *C. pipiens* mosquitoes in the offspring generation as a function of temperature and pesticide treatments in the parental (F0) and offspring (F1) generations. Development times are based on 9 replicate insectaries per treatment combination. Given are LS-means with 1 SE. The asterisks indicate significant effects of warming for a given pesticide treatment (* *P* < 0.05, ** *P* < 0.01, *** *P* < 0.001)


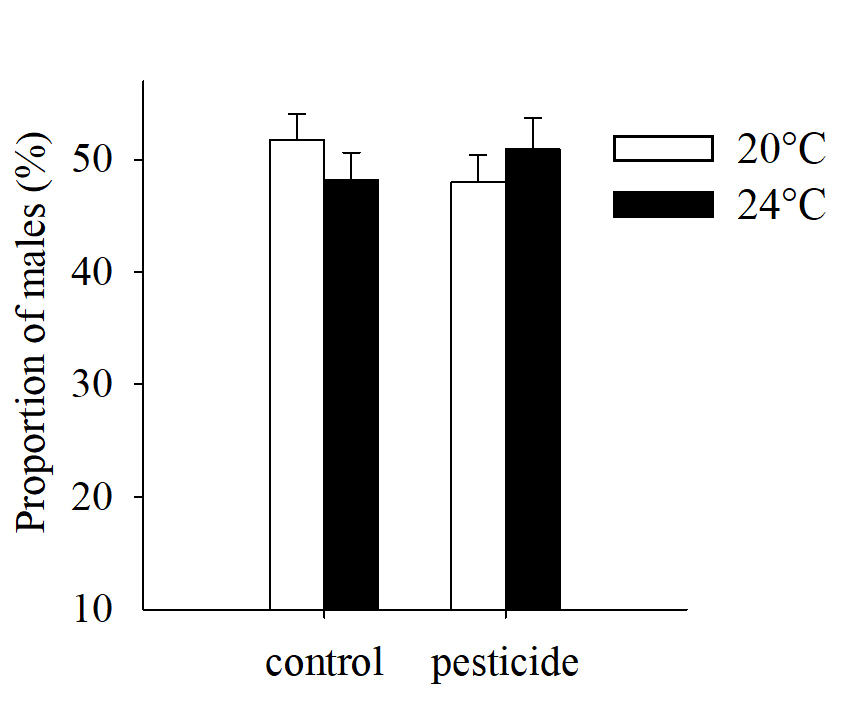


Figure S5. Proportion of male *Culex pipiens* in the parental generation as a function of temperature and pesticide treatments. Proportion of males is based on 9 replicated insectaries per treatment combination.


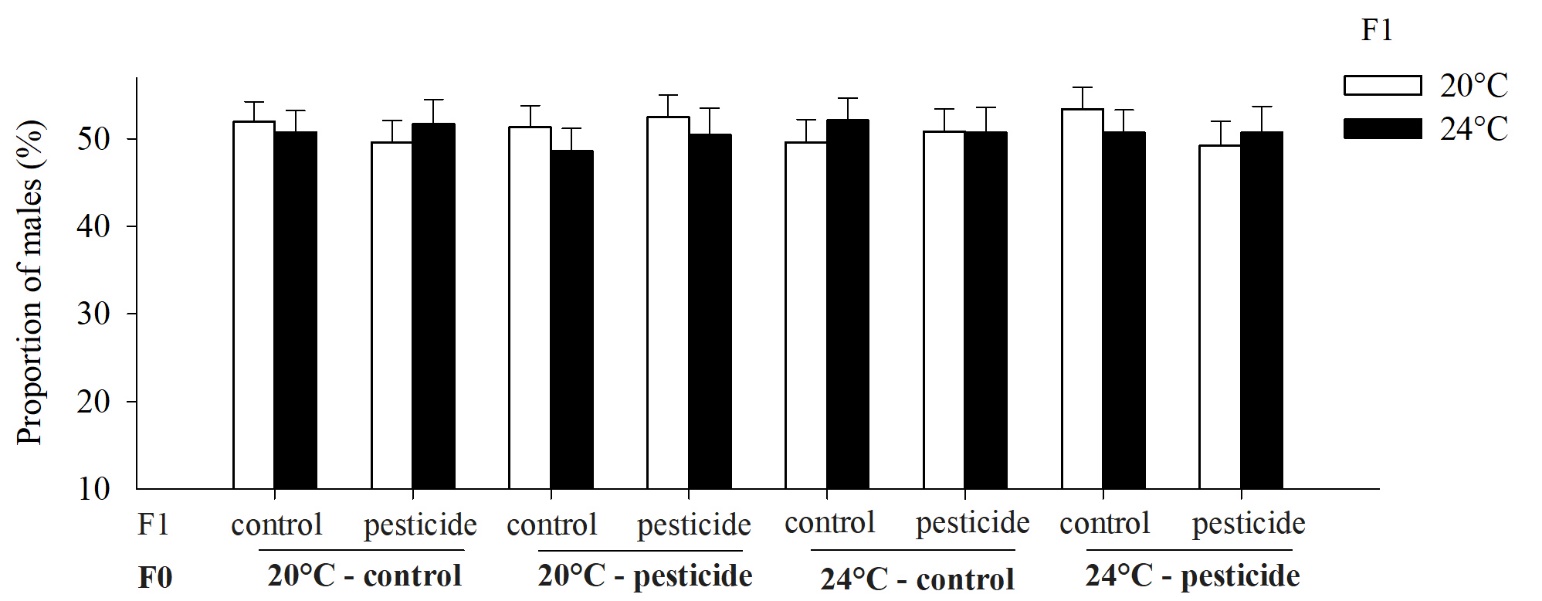


Figure S6. Proportion of male *Culex pipiens* in the offspring generation as a function of temperature and pesticide treatments in the parental (F0) and offspring (F1) generations. Proportion of males is based on 9 replicated insectaries per treatment combination.
