# Supplementary material for: Transgenerational interactions between pesticide exposure and warming in a vector mosquito
Source: Evol Appl. 2018 Mar 5;11(6):906–17. doi: 10.1111/eva.12605 (PMC5999214; doi:10.1111/eva.12605)
Supplement: Supplementary file 3 [file EVA-11-906-s003.docx]

**Appendix 3. Full models and figures including the effect of sex on size at emergence in both generations**

In both generations, size at emergence of the mosquitoes was significantly different between males and females with females being larger (Table S3, Fig. S7 & S8). This confirms results of previous studies ([Mahajan et al. 2011](#_ENREF_1); [Op de Beeck et al. 2016](#_ENREF_2); [van Uitregt et al. 2012](#_ENREF_3)).

Table S3. Effects of temperature, pesticide exposure and sex on size at emergence of *Culex pipiens* mosquitoes in the parental generation (F0) and in the offspring generation (F1).

| Effect | Size at emergence (F0) | | | Size at emergence (F1) | | |
| --- | --- | --- | --- | --- | --- | --- |
|  | df1 | χ^2^ | *P* | df1 | χ^2^ | *P* |
| Temperature F0 (Temp F0) | 1 | 113.692 | **<0.001** | 1 | 2.40 | 0.122 |
| Pesticide F0 (Pest F0) | 1 | 0.034 | 0.855 | 1 | 0.27 | 0.603 |
| Temp F0 × Pest F0 | 1 | 0.007 | 0.932 | 1 | 2.61 | 0.106 |
| Temperature F1 (Temp F1) |  |  |  | 1 | 752.62 | **<0.001** |
| Pesticide F1 (Pest F1) |  |  |  | 1 | 18.32 | **<0.001** |
| Temp F0 × Temp F1 |  |  |  | 1 | 2.70 | 0.100 |
| Pest F0 × Temp F1 |  |  |  | 1 | 0.06 | 0.814 |
| Temp F0 × Pest F1 |  |  |  | 1 | 2.30 | 0.129 |
| Pest F0 × Pest F1 |  |  |  | 1 | 6.34 | **0.012** |
| Temp F1 × Pest F1 |  |  |  | 1 | 1.53 | 0.215 |
| Temp F0 × Pest F0 ×Temp F1 |  |  |  | 1 | 0.14 | 0.705 |
| Temp F0 × Pest F0 × Pest F1 |  |  |  | 1 | 2.16 | 0.142 |
| Temp F0 × Temp F1 × Pest F1 |  |  |  | 1 | 0.06 | 0.814 |
| Pest F0 × Temp F1 × Pest F1 |  |  |  | 1 | 0.05 | 0.823 |
| Temp F0 × Pest F0 × Temp F1 × Pest F1 |  |  |  | 1 | 0.21 | 0.644 |
| Sex | 1 | 1718.826 | **<0.001** | 1 | 11091.99 | **<0.001** |
| Temp F0 × Sex | 1 | 6.489 | 0.011 | 1 | 4.39 | **0.036** |
| Pest F0 × Sex | 1 | 1.783 | 0.182 | 1 | 16.15 | **<0.001** |
| Temp F0 × Pest F0 × Sex | 1 | 2.964 | 0.085 | 1 | 3.68 | 0.055 |
| Temp F1 × Sex |  |  |  | 1 | 0.16 | 0.688 |
| Pest F1 × Sex |  |  |  | 1 | 0.23 | 0.268 |
| Temp F0 × Temp F1 × Sex |  |  |  | 1 | 2.15 | 0.142 |
| Pest F0 × Temp F1 ×Sex |  |  |  | 1 | 3.17 | 0.075 |
| Temp F0 × Pest F1 ×Sex |  |  |  | 1 | 0.05 | 0.823 |
| Pest F0 × Pest F1 × Sex |  |  |  | 1 | 2.60 | 0.106 |
| Temp F1 × Pest F1 × Sex |  |  |  | 1 | 1.18 | 0.278 |
| Temp F0 × Pest F0 × Temp F1 × Sex |  |  |  | 1 | 0.770 | 0.380 |
| Temp F0 × Pest F0 × Pest F1 × Sex |  |  |  | 1 | 2.06 | 0.113 |
| Temp F0 × Temp F1 × Pest F1 × Sex |  |  |  | 1 | 1.25 | 0.264 |
| Pest F0 × Temp F1 × Pest F1 × Sex |  |  |  | 1 | 2.29 | 0.130 |
| Temp F0 × Pest F0 × Temp F1× Pest F1 × Sex |  |  |  | 1 | 1.84 | 0.168 |

Significant *P* values (*P* < 0.05) are indicated in bold.


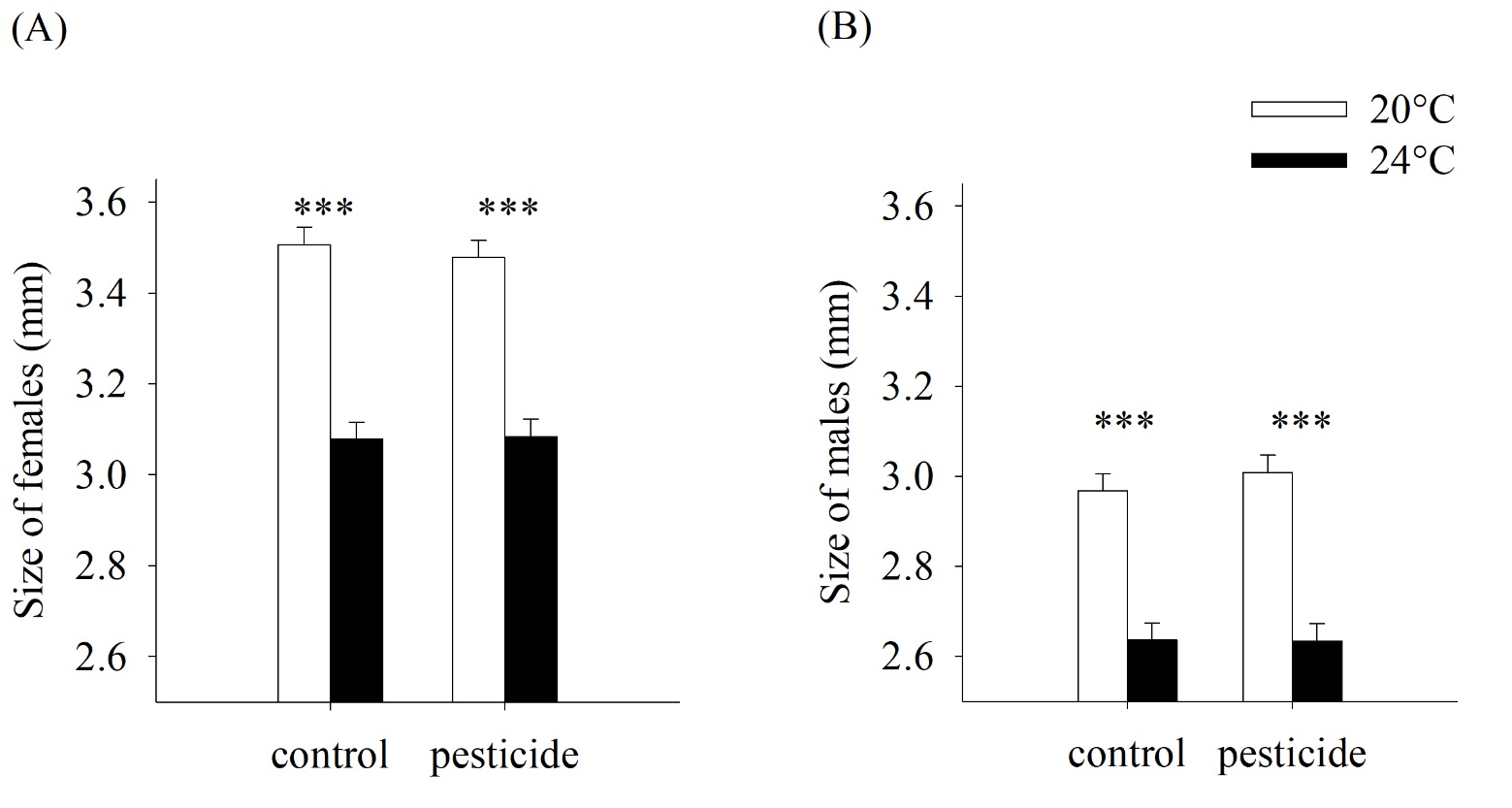


Figure S7 Size at emergence of the adults in the parental generation (A: females, B: males) as a function of temperature and pesticide treatment. Given are LS-means with 1 SE. The asterisks indicate significant effects of warming for a given pesticide treatment (* P < 0.05, ** P < 0.01, *** P < 0.001). There were 9 insectary replicates for all treatment combinations.


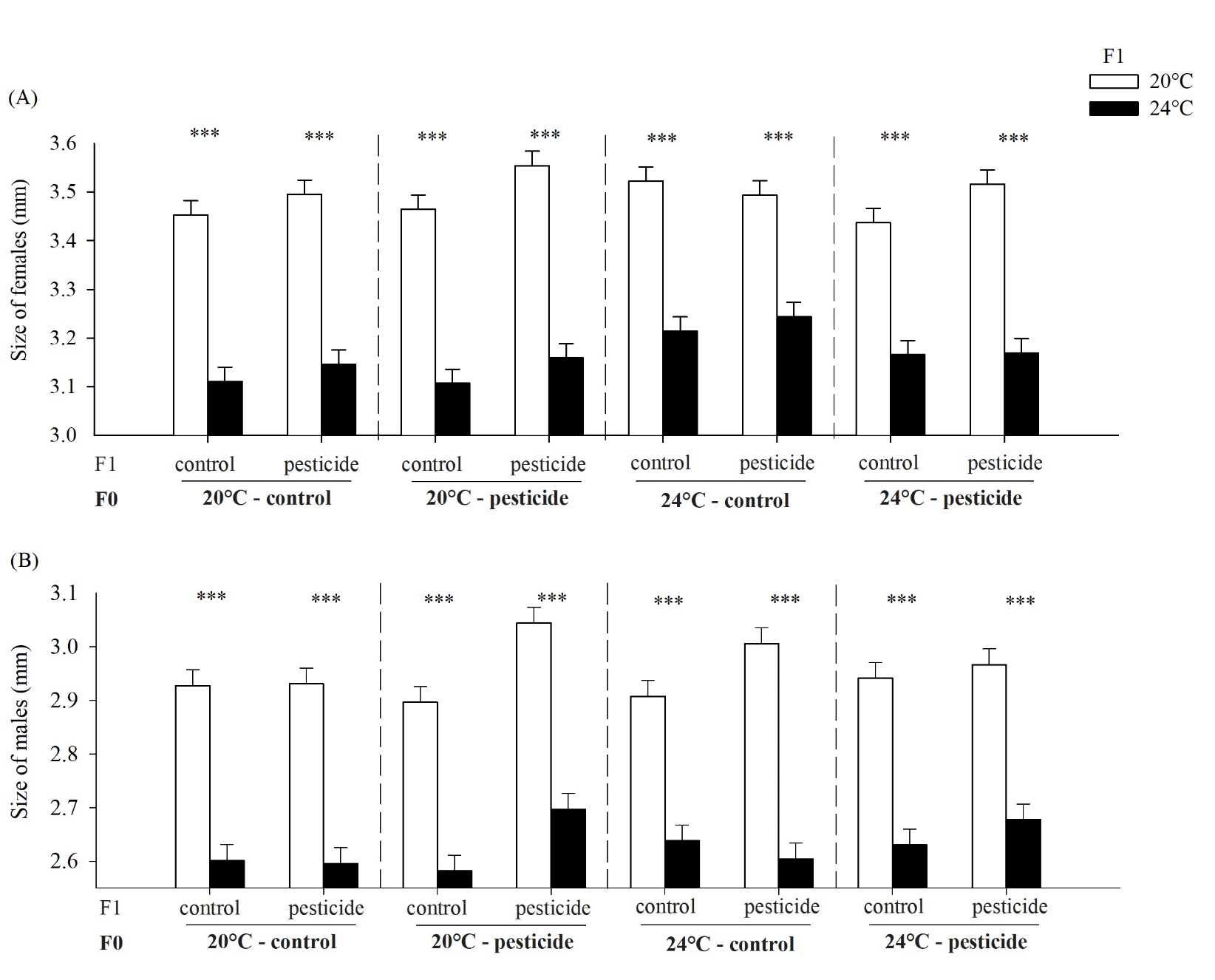


Figure S8. Size at emergence of the offspring (A: females, B: males) as a function of temperature and pesticide treatment in both generations. Given are LS-means with 1 SE. The asterisks indicate significant effects of warming for a given pesticide treatment (* *P* < 0.05, ** *P* < 0.01, *** *P* < 0.001). There were 9 insectary replicates for all treatment combinations.

**Literature cited**

Mahajan, U. V., J. Gravgaard, M. Turnbull, D. B. Jacobs, and T. L. McNealy. 2011. Larval exposure to *Francisella tularensis* LVS affects fitness of the mosquito *Culex quinquefasciatus*. FEMS Microbiology Ecology **78**:520-530.

Op de Beeck, L., L. Janssens, and R. Stoks. 2016. Synthetic predator cues impair immune function and make the biological pesticide Bti more lethal for vector mosquitoes. Ecological Applications **26**:355-366.

van Uitregt, V. O., T. P. Hurst, and R. S. Wilson. 2012. Reduced size and starvation resistance in adult mosquitoes, *Aedes notoscriptus*, exposed to predation cues as larvae. Journal of Animal Ecology **81**:108-115.
