# Supplementary figures and images for: Transgenerational interactions between pesticide exposure and warming in a vector mosquito
Source: Evol Appl. 2018 Mar 5;11(6):906–17. doi: 10.1111/eva.12605 (PMC5999214; doi:10.1111/eva.12605)

# PARENTS (F0)

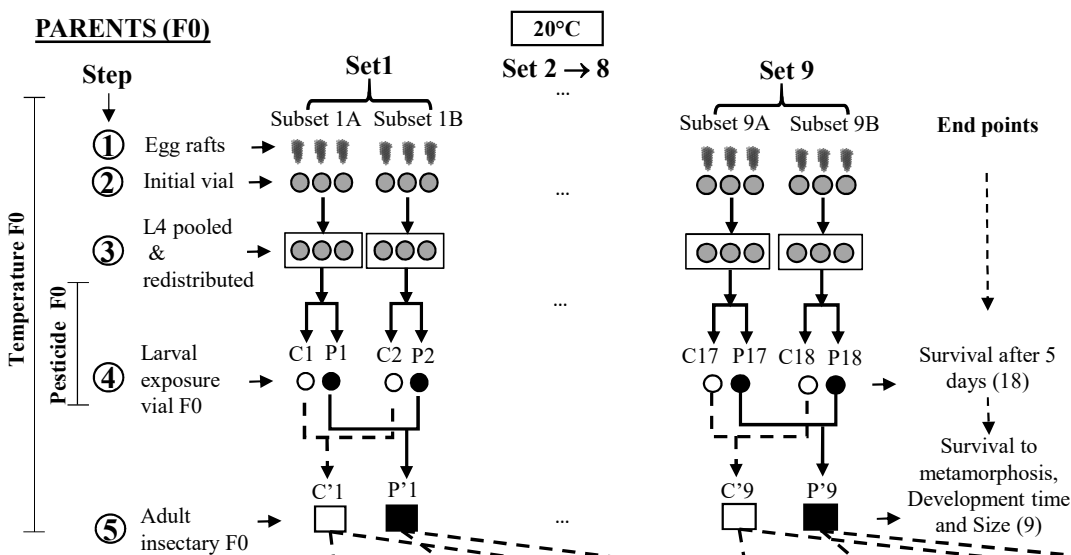

# OFFSPRING (F1)

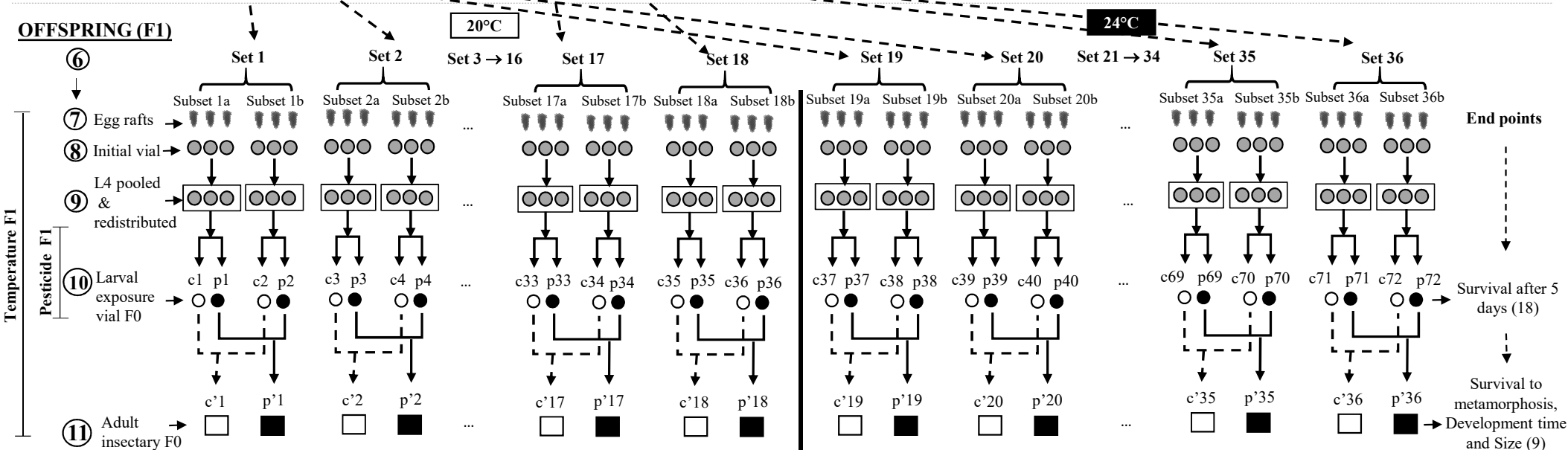

Supplement: Supplementary file 4 [file EVA-11-906-s004.pdf]

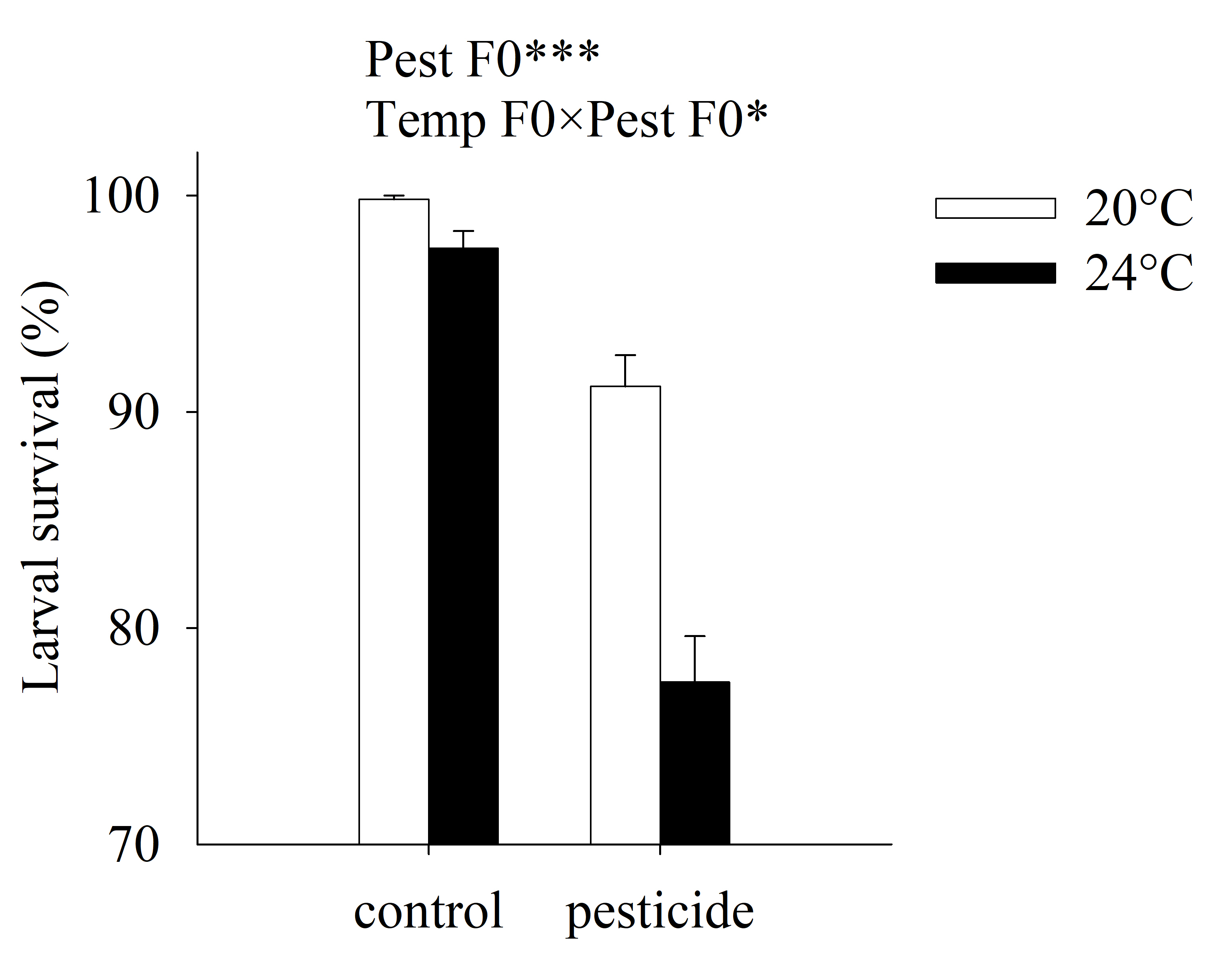

Supplement: Supplementary file 5 [file EVA-11-906-s005.JPG]

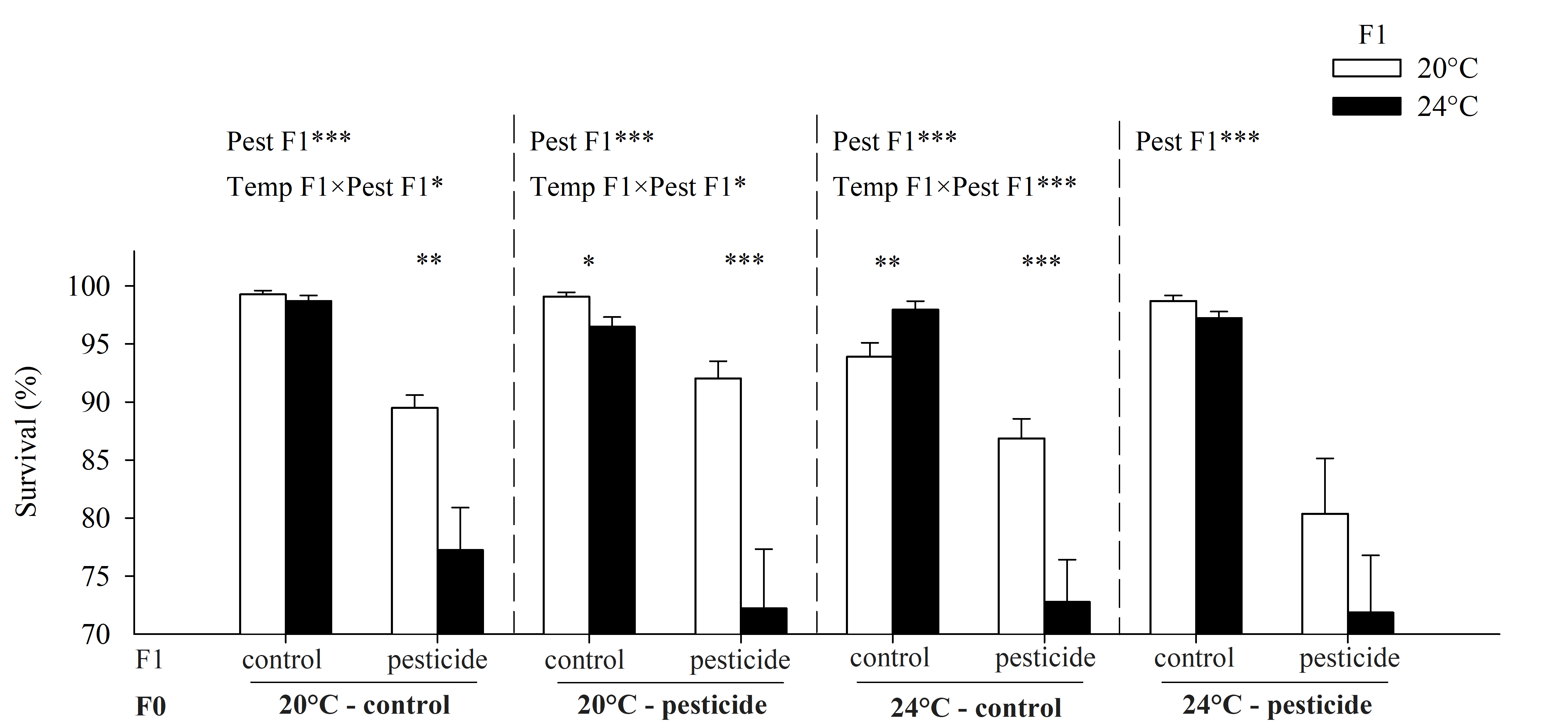

Supplement: Supplementary file 6 [file EVA-11-906-s006.JPG]

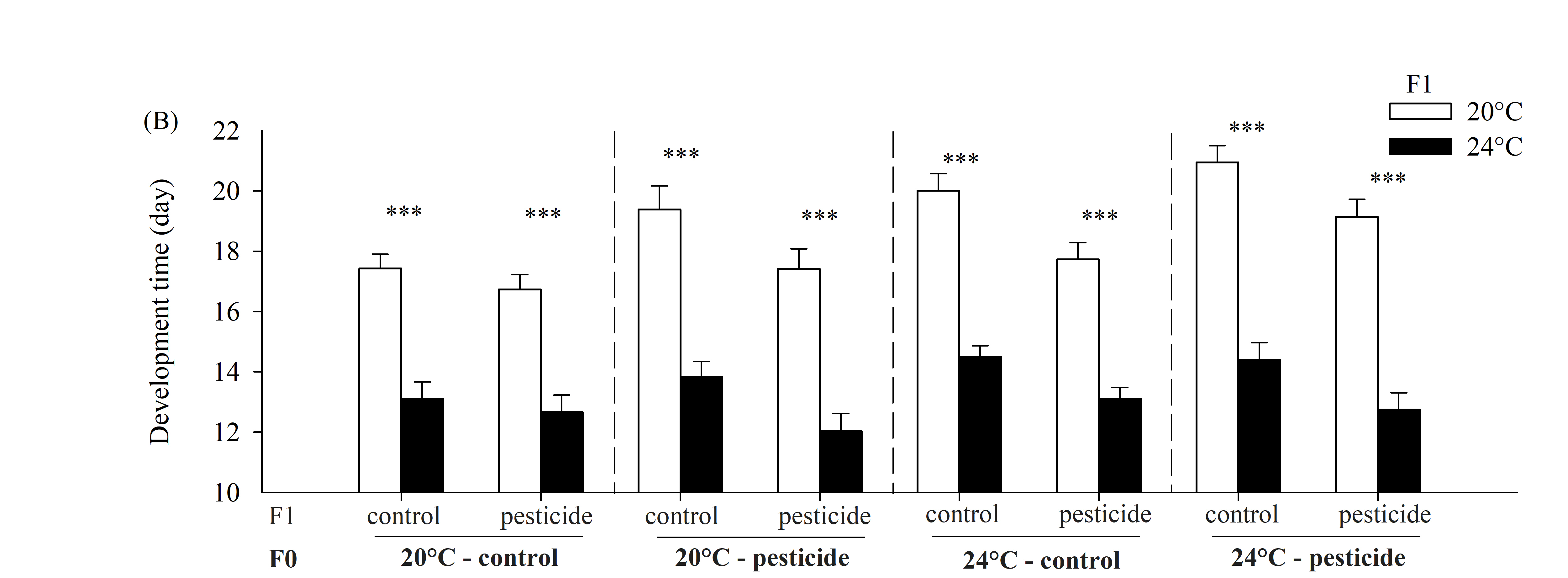

Supplement: Supplementary file 7 [file EVA-11-906-s007.JPG]

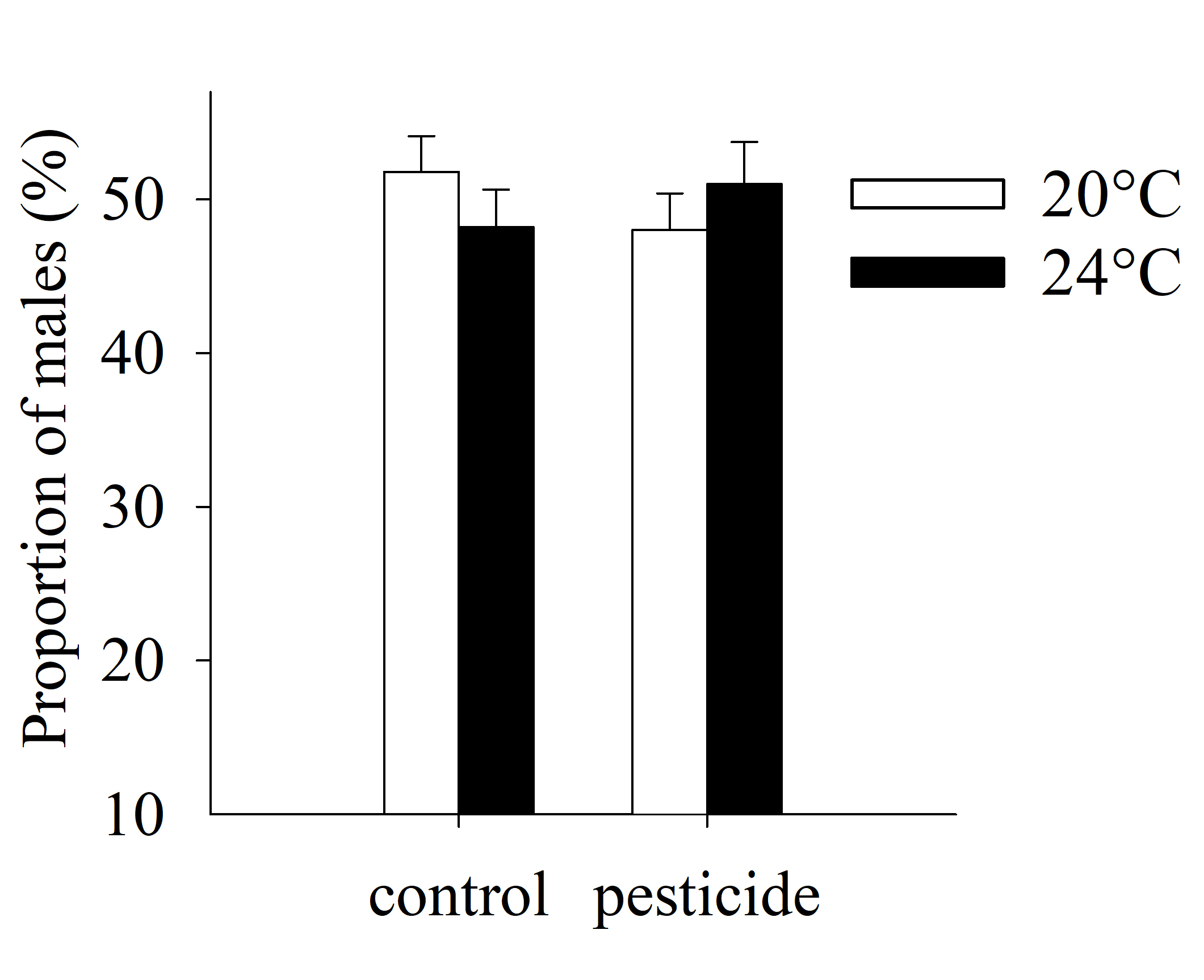

Supplement: Supplementary file 8 [file EVA-11-906-s008.JPG]

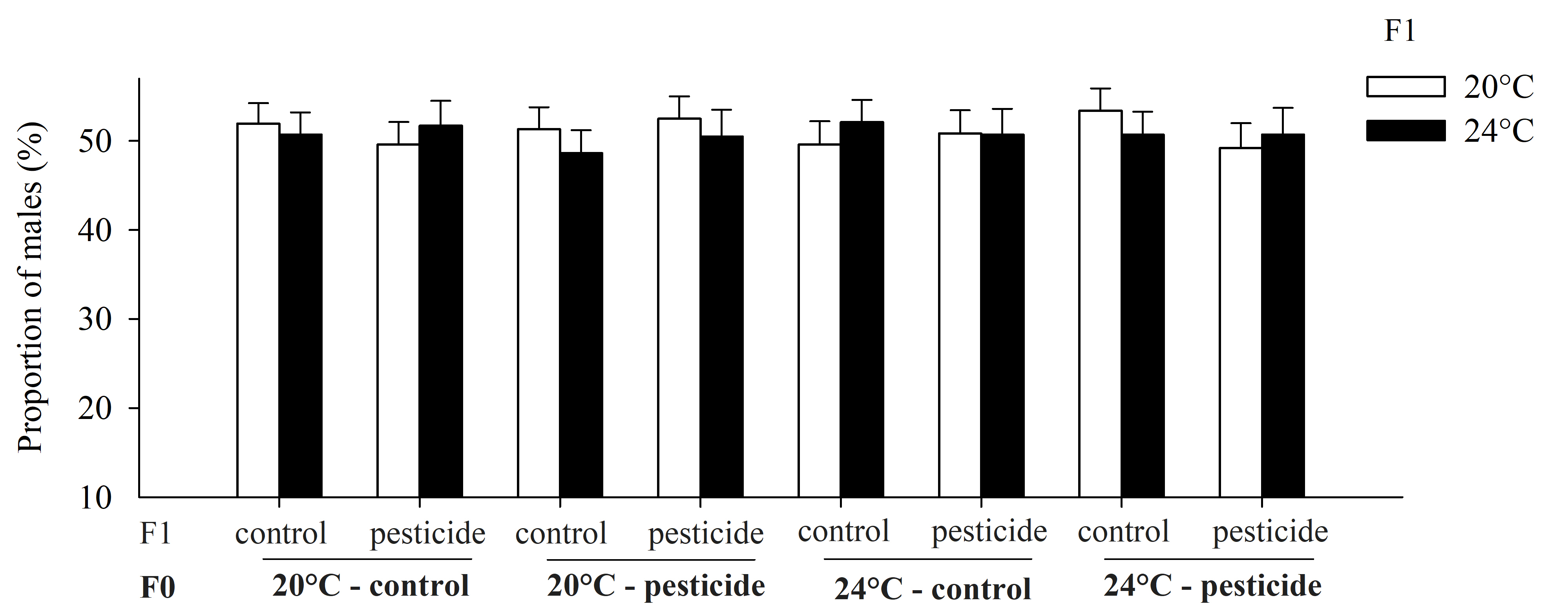

Supplement: Supplementary file 9 [file EVA-11-906-s009.JPG]
